# Insight and Recent Advances into the Role of Topography on the Cell Differentiation and Proliferation on Biopolymeric Surfaces

**DOI:** 10.3390/ijms23147731

**Published:** 2022-07-13

**Authors:** Raluca Tudureanu, Iuliana M. Handrea-Dragan, Sanda Boca, Ioan Botiz

**Affiliations:** 1 Interdisciplinary Research Institute in Bio-Nano-Sciences, Babeș-Bolyai University, 400271 Cluj-Napoca, Romania; raluca.tudureanu@ubbcluj.ro (R.T.); iuliana.dragan@ubbcluj.ro (I.M.H.-D.); sanda.boca@ubbcluj.ro (S.B.); 2 Faculty of Physics, Babeș-Bolyai University, 400084 Cluj-Napoca, Romania

**Keywords:** biopolymeric films, lithographic methods, surface relief patterns, cell differentiation, cell proliferation

## Abstract

It is well known that surface topography plays an important role in cell behavior, including adhesion, migration, orientation, elongation, proliferation and differentiation. Studying these cell functions is essential in order to better understand and control specific characteristics of the cells and thus to enhance their potential in various biomedical applications. This review proposes to investigate the extent to which various surface relief patterns, imprinted in biopolymer films or in polymeric films coated with biopolymers, by utilizing specific lithographic techniques, influence cell behavior and development. We aim to understand how characteristics such as shape, dimension or chemical functionality of surface relief patterns alter the orientation and elongation of cells, and thus, finally make their mark on the cell proliferation and differentiation. We infer that such an insight is a prerequisite for pushing forward the comprehension of the methodologies and technologies used in tissue engineering applications and products, including skin or bone implants and wound or fracture healing.

## 1. Introduction

The behavior of a cell depends on a variety of internal and external factors, such as growth factors [1], hormones, adhesion factors [2] and the extracellular matrix [3]. The “destiny” of a cell is controlled by the signals it receives from the other surrounding cells through the extracellular environment [4]. For instance, when performing in vitro cell cultures, the substrates onto which cells are grown have to possess some specific properties [4,5], including topography, elasticity, gradients [6], biocompatibility, and hydrophobicity [7]. Therefore, it is important to understand the impact of the physical and chemical properties of a substrate on various cells in order to optimize their culture and control their differentiation and proliferation. Because the cell culture is a highly important process in domains like tissue engineering, regenerative medicine [8], and biotechnology [9], the cell culture techniques have rapidly flourished and evolved [10], and can be now performed in suspensions and on adherent surfaces, including scaffold-based techniques (also called 3D cultures) using hydrogel-based support, polymeric hard material-based support or hydrophilic glass fibers [11].

There is a significant number of studies related to controlling the cell orientation [12], proliferation [13], differentiation [6,7] and adhesion [11,14] through the employment of different types of substrates covered with specific layers of (bio)polymers, eventually patterned with miniaturized (periodic) surface relief patterns [5,11,15,16,17]. The latter can be created by utilizing a variety of simple or more complex lithography methods, ref. [18,19,20,21] and may include grooves [22], lines [23,24,25], squares [23,26] and other shapes [13,27,28,29] of various dimensions and functions widely used in applications related inclusively to cell culturing [30,31,32,33,34,35]. For instance, holes and pillars on gelatin-genipin substrates can be used for culturing human osteoblastic Saos-2 cells for developing better properties of surface dental implants [22], and the microgrooves on the chitosan membrane can be employed for enhancing nerve regeneration using Schwan cell cultures [36].

Considering the need for better strategies to improve the cell growth and development processes, the use of periodic surface relief structures as support substrates for cell cultures might be a part of the solution, as relief structures can be made of cell compatible material and can exhibit cell dimensions. Such novel and improved materials have a significant potential not only to provide a better understanding of processes of cell proliferation and differentiation, but also to improve and develop new cell culture techniques. Herein, we review the most recent approaches that utilize surface relief structures to push the limits of cell culture, differentiation and proliferation beyond the state-of-the art. A short description of the most popular methods employed to pattern (bio)polymers is firstly presented, followed by a general part containing basic information about cell proliferation and differentiation, a brief enumeration of the main microscopic techniques used to study the above-mentioned processes, and a last part that exemplifies the role and impact of surface relief patterns on the cell proliferation and differentiation processes.

## 2. Common Lithographic Methods Used for Biopolymer Patterning

There is a tremendous amount of periodic surface relief patterns exhibiting a certain shape, dimension and function [37,38,39,40,41,42,43]. Such periodic patterns are mainly obtained from polymer-based systems (generally, polymers are cheap and yet highly processable materials) [44,45,46,47], through the use of various top-down and bottom-up methodologies [21,48]. Because relief patterns can be filled with multifunctional materials of different size and shape, a variety of multifunctional platforms can be developed and used in a plethora of applications [21], including biological ones [42,49,50]. Biopolymeric surface relief patterns represent an interesting category of relief patterns, as they can be employed to design puzzling experiments in cell growth [13] or at the biology-electronics interface [43], and can be obtained by sculpturing biopolymeric films [13,18,19,20,51,52]. For instance, through the use of photolithography, porous films of biopolymers such as chitosan, starch and their blend can be obtained with the aid of light, by inducing specific chemical changes that allow the (bio)photoresist to be removed later on [13]. The transfer of the surface relief patterns can be realized either by the direct laser writing (DLW) or through the exposure of the photoresist to (extreme) UV light and the additional use of a photomask. As a result, precise shape-defined particles of silk [18] or keratin proteins [19] can be fabricated. Furthermore, the patterning of silk proteins via photolithography can provide substrates covered with complex micro spatial morphologies that are highly suitable for many medical applications, including drug delivery, tissue engineering and degradable implants [53,54].

Electron (EBL) and ion beam (IBL) lithographies can also write high resolution patterns onto biopolymer-based resists by utilizing focused beams of electrons and ions, respectively [20]. While the electrons change the solubility of the resist and allow selective removal of exposed or nonexposed regions by subsequent etching in a solvent, ions might be used to remove parts of biopolymer-based resists. For both techniques, the resist type and quality are highly important [43,55,56] when beautiful lines/spacing gratings, moth-eye patterns, or pads are to be fabricated in sugar-based polymers, biotinylated polyethylene glycol (PEG), DNA oligonucleotides, neutravidin, anti-mouse IgG [41,43,57]. More details on biopolymers-based patterns created using EBL and IBL can be found in the literature [20].

**Figure 1:**
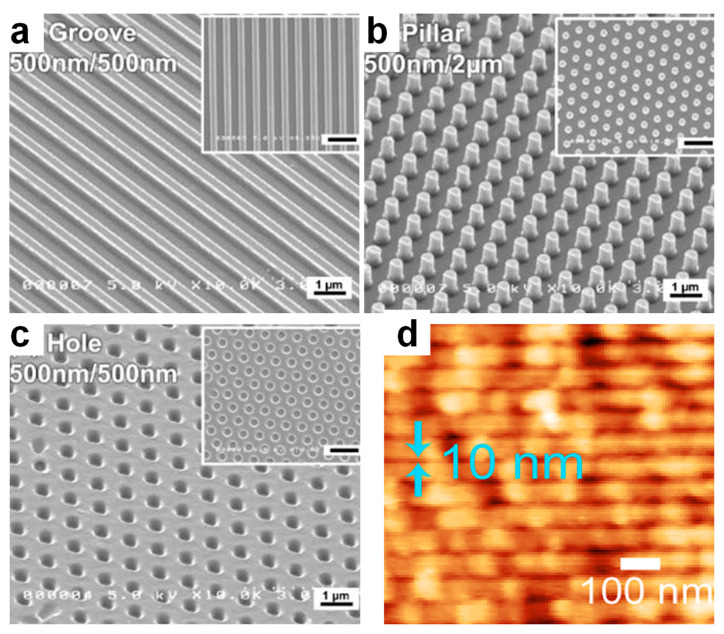
SEM images of different biopolymer-based patterns: (**a**–**c**) Cross-section and top (insets) SEM images emphasizing grooves (**a**), pillars (**b**) and holes (**c**) of gelatin crosslinked with genipin. The scale bar in the insets corresponds to 2 µm. (**d**) AFM topography micrograph depicting high resolution single-line enzyme patterns made on a copolymer and displaying a width smaller than 10 nm. Adapted with permission from ref. [22] (**a**–**c**) and ref. [23] (**d**).

High-quality biopolymeric patterns of sub-micrometer dimensions [58] can be further obtained by employing soft lithography. This technique uses soft/elastomeric molds to replicate various periodic surface relief patterns over relatively large areas [59] either by a fast transfer of a model pattern from a master mold to a biopolymeric resist through the mechanical deformation upon thermal treatments and UV illumination or through the conformal contact of specific polymeric surfaces with a master stamp exhibiting model patterns covered with biopolymer-based inks. While the former is called nanoimprint lithography (NIL), the latter is known as microcontact printing (μCP). As a result, there is a plethora of miniaturized periodic biopolymeric patterns, of which the most popular are lines/stripes of various proteins, chitosan, cellulose, silk and other biomolecules [24,25,27,58,60,61]. Other patterns include pads of biomolecules [27], nanodots of proteins [62] as well as pillars, grooves and holes sculptured in cellulose, and in gelatins crosslinked with genipin (Figure 1a–c) or in chitosan [22,24,28,29]. More complex patterns such as spider web arrays [61] and arrays of nanodots of neutravidin, liposomes and other proteins [62,63] were also recently reported. Additional relevant information can be found in the literature [64].

Highly miniaturized biopolymeric relief patterns can also be developed via mold-free scanning probe lithography (SPL) through the utilization of extremely sharp scanning probes capable to exert stimuli such as force, heat, or electric fields, to induce chemical reactions or to diffuse ink molecules. For example, SPL can generate periodic patterns such as lines, dots, rectangles or squares of enzymes on specific copolymers [23,65]. Some of these patterns, such as the sub-10 nm lines, closely match the enzyme molecular dimension and could be used in the fabrication of various biodevices [23,65] (Figure 1d). Other types of patterns of biopolymers include protein covered circular patches of a diameter ranging from 15 nm to 200 nm that can be realized using particle lithography (PL) [66]. This method is based on the use of a particle mask, which is usually assembled from silica or various polymeric particles on top of a solid substrate. More details on these patterning techniques can be found elsewhere [21].

Besides the top-down lithographic methodologies, bottom-up approaches can also be used to fabricate miniaturized biopolymeric surface relief patterns, especially by taking advantage of DNA self-assembly (DNASA). With this approach, sub-10 nm patterns [67], often displaying a wide range of shapes that include squares, rectangles, stars, disks, five-pointed stars, or triangles, can be obtained [26,67,68]. When needed, DNA nanostructures can be further combined with other top-down approaches to create nanopatterns that exhibit significant potential in biomolecular recognition [68] or in ultrascaled technology nodes [69]. Combining DNASA with IBL can lead to peculiar 10-nm sized DNA-based surface relief patterns such as arrays of DNA origami immobilized in IBL patterns [49]. Other shapes of individual DNA within specific surface relief patterns can be obtained as well [69,70]. For instance, specific DNA bricks can be assembled to form DNA brick crystal-based periodic nanotrenches displaying a uniform pitch down to nanoscale [69]. For more detail on the variety of possible biopolymeric relief patterns, readers should consult Table 1 and reference [21].

## 3. Description of Cell Proliferation and Differentiation Processes

### 3.1. Basics of Cell Proliferation

Cell proliferation is an essential process with the key role of maintaining the life of an organism through the healing of wounds, developing the organs and regenerating the tissues [71]. To accurately perform these tasks, the cells must receive specific signals that control their division and apoptosis [72]. Otherwise, if the downregulated growth does not take place, the impact of cell proliferation is harmful and life-threatening (e.g., in pathologies like cancers [71]). Hence, the biochemical, molecular and bioelectric mechanisms that regulate the cell cycle [71] ensure that cell growth is in accordance with the needs of the organism [71,73].

Generally, for eukaryotic cells there are two types of cell divisions: mitosis, whereby each daughter cell is genetically identical to the parent cell, and a reproductive cell division (meiosis), in which the number of chromosomes in the daughter cells is reduced by half to produce haploid gametes [74]. A somatic cell divides through the mitosis process that takes place in four phases: First gap (G1), synthesis (S), second gap (G2) and mitosis (M) [75] (although there are also cases when a cell enters in a non-dividing status, the G0 phase [71,76]). For each of these phases there are specific mechanisms of cell regulation [77,78]. Thus, depending on different factors such as nutrients or mitogens, a cell can go through G1 phase and activate a program that consists in next steps of cell division or enter in a quiescent phase, G0 [78], which is a resting state until new signals are received to stimulate a further entering into the cell division cycle or not [79]. Relevant details on mechanisms that signal a cell to enter into the G1 phase with the help of various proteins can be found in the literature [76,77,80,81].

While in the G1 phase, cells prepare to replicate their DNA and therefore, mRNA and protein required for DNA development are synthesized [76], in phase S the quantity of the genetic material DNA is continuing to increase and eventually doubles [75,76,80,81], turning a 2N complement (i.e., 2 copies of each chromosome) of DNA cells to a 4N complement of DNA cells. This means that the initial haploid cell turns into a diploid cell towards the end of the S phase [75]. Instead, the G2 phase is mostly a verification step in the cell cycle, when a check for any DNA damage that may have occurred in the replication process takes place [76]. Finally, while the pre-mitosis steps are very important for ensuring the integrity and health of the resulting cells [76], the mitosis phase is the part of the somatic cellular division where the duplicated genetic material is equally divided between the two progeny cells that will separate at the end into two daughter cells [75].

**Figure 2:**
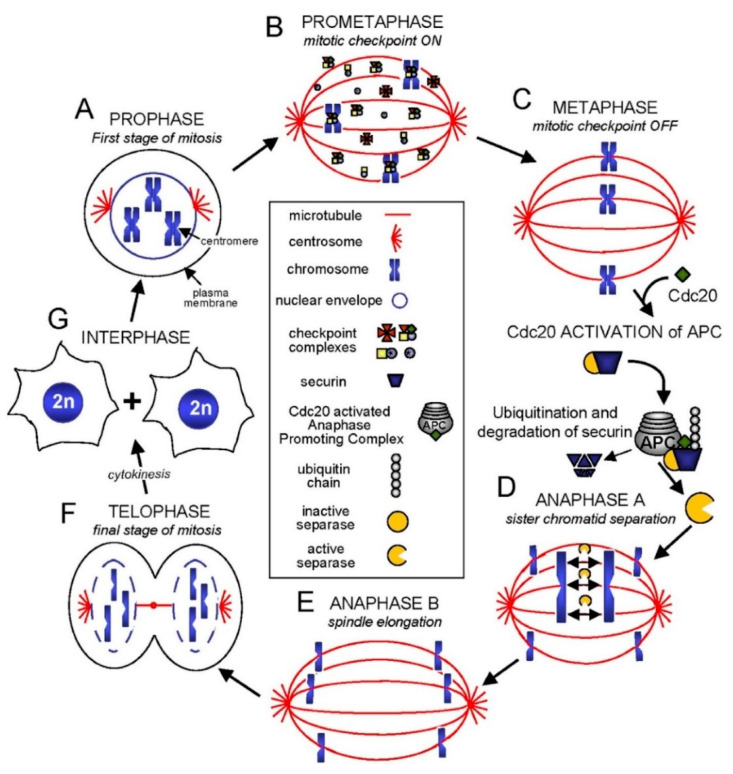
Schematics depicting the phases of mitosis. Reproduced with permission from ref. [86].

The mitosis phase itself is divided into five phases (Figure 2): prophase, prometaphase, metaphase, anaphase and telophase [82,83]. In prophase, the chromatin (i.e., DNA that is wrapped around some proteins named histones) is firstly condensed and then followed by the ending of the DNA transcription and dispersion of the nucleus envelope. This dispersion allows the nucleoplasm to mix with cytoplasm where new, more labile microtubules (MTs) form [82] along with actin filaments [84] (prometaphase). While some MTs will structure the division spindle (i.e., the cytoskeletal structure), the actin filaments will be utilized in the process of cytokinesis (i.e., the formation of a cleavage furrow that divides the cell membrane into half and separates the two daughter cells). Specific to the metaphase is the alignment of the chromosomes on the spindle midplane, forming the “metaphase plate” [75,82]. At the same time, the bonds between chromatids dissolve and allow the next process of division (the anaphase) to begin. In this phase, each of the two chromatids that form the chromosomes in the metaphase becomes a chromosome. While the resulting chromosomes will migrate to the extremity poles of the cell (Figure 2) [75,82,85], the connecting fibers shorten and the spindle elongates causing the separation of the two poles from each other [85]. Once the chromosomes are reaching the poles, the telophase begins and the nuclear membranes start to form. They will envelop each of the two sets of chromosomes. The later start to decondense and the spindle begins to disassemble. The division ends with the process of karyokinesis when the two daughter cells separate. Both cells are now ready to enter in the interphase and eventually the G1 phase restarts [82].

### 3.2. Basics of Cell Differentiation

**Figure 3:**
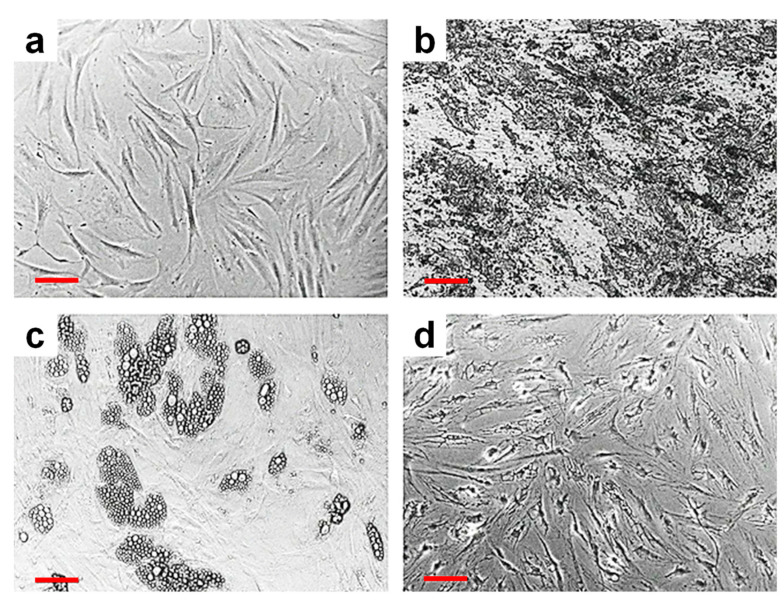
Human adipose MSCs undifferentiated cells (**a**) suffering osteogenic (**b**), adipogenic (**c**) and neurogenic (**d**) differentiation, respectively. Scale bars in red represent 100 µm. Adapted with permission from ref. [96].

Stem cells are a particular type of cells that have two important properties: self-renewal and developing into different specialized functional cells [87,88,89]. According to their potential to differentiate, there are three types of stem cells: totipotent (TSCs), pluripotent (PSCs) and multipotent (MPSCs) [87,90]. There are also unipotent cells (USCs), but those have a very low capacity of potency, being able to differentiate only in one type of cells, depending on the tissue where they are found [91]. While TSCs are the cells that result from the fusion of the sperm cell and the oocyte and by division they form the embryonic and extraembryonic cells [87], PSCs have the ability to form the ectoderm, mesoderm and endoderm, which are the three germ layers that lately can differentiate into different categories of functionalized cells [92], giving rise to system organs [87]. Therefore, PSCs can be found only in the early stages of embryonic development [91]. Nonetheless, induced pluripotent stem cells (iPSCs), i.e., reprogrammed multipotent stem cells that become pluripotent cells, can be obtained via a process described by Takahashi and Yamanaka [93] and represent an important turning point in stem cells studies and therapies since 2006 [93,94]. Meanwhile, MPSCs cells give rise to cells with the same particular properties like hematopoietic stem cells (HSCs), mesenchymal stem cells (MSCs) and neural stem cells (NSCs). Furthermore, while HSCs can differentiate into all types of blood cells such as lymphoid cells (natural killer cells, B- and T-lymphocytes) and myeloid cells (erythrocytes, platelets, neutrophils, basophils, eosinophils, monocytes and macrophages) [95], MSCs have the capacity to differentiate into connective tissue cell types [91,96] (osteocytes, adipocytes, chondrogenic cell lineages) (Figure 3) [91].

Furthermore, there are two broad types of stem cells according to their developmental stage: Embryonic stem cells (ESCs) and adult stem cells (ASCs) [87]. While situated in the epiblast tissue and the inner wall of the blastocyst, a structure that forms during the fifth day of human embryo development, ESCs can develop in more than 200 types of adult cells depending on the specific signaling factors [87]. The most common expanded stem cells in culture are ESCs, ASCs and iPSCs [97]. Generally, ESCs are able to self-renew indefinitely under the appropriate conditions, although the possibility of the development of some karyotypic abnormalities through the culture passages cannot be excluded [98]. In this context, the type of the cell culture medium that not only feeds the cells, but also works as an instructor for the cell fate plays a critical role. Moreover, due to the existence of a huge variety of stem cells, it is difficult to use only a certain type of cell culture medium that suits the needs of every type of stem cell [97]. In this regard, special attention was given to the development of a culture medium type that contains specific chemical compounds for maintaining the undifferentiated form of cells [99], including βTGF and FGF [100], fetal calf serum [101] or others [102].

## 4. Microscopic Techniques for Cell Proliferation and Differentiation Assessment and Observation

The most common microscopic techniques used to follow the processes of cell differentiation and proliferation are brightfield/fluorescence microscopy [103] and scanning electron (SEM) microscopy [104]. Brightfield microscopy is usually sufficient to see the general outlines of cells, but to achieve detailed, high-contrast images, phase contrast or differential interference contrast (DIC), which gives a pseudo–three-dimensional (3D) shaded appearance to cells, is necessary. Regarding fluorescence microscopy, the confocal configuration appears to be optimal to follow cells, although the utility of other techniques such as two-photon excitation microscopy [105], widefield fluorescence microscopy, and total internal reflection fluorescence microscopy [106] has been also demonstrated.

**Figure 4:**
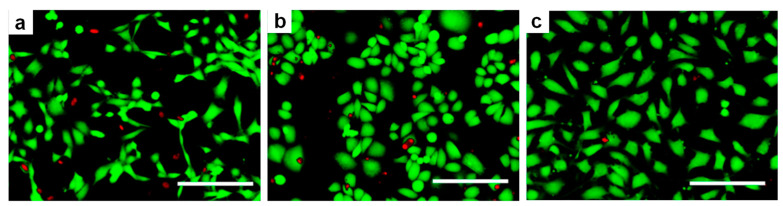
(**a**–**c**) Fluorescent micrographs of 3T3 fibroblasts (**a**), HaCat keratinocytes (**b**) and EA.hy926 endothelial (**c**) cells cultured on PHBV-GelMA patches. Scale bars: 200 μm. Adapted with permission from ref. [113].

Confocal microscopy is an optical non-destructive imaging method used to record both 2D and 3D microscopic images of topography and morphology of various biopolymeric substrates and biological samples [103,107] that focuses a small beam of light in depth of field by using confocal pinholes [108]. This configuration allows not only the obtaining of high resolution images of the surroundings of the surface structures, but also to estimate the thickness of such structures [107]. To increase the contrast of the desired samples, the fluorescence confocal microscopy system uses fluorochromes such as photocleavable proteins [52] on protein-based materials and on stromal tissues, neoplastic breast and ductal carcinoma samples [109]. Moreover, confocal microscopy can be used not only to identify surface irregularities, roughness or more evident relief patterns [103,107,110,111], but it can also be employed in cell studies of (i) human umbilical endothelial cells on interpenetrating polymer network hydrogel [112], (ii) of rat bone marrow MSCs on micro arrays of poly (ethylene glycol)-poly(ε-caprolactone) [20], (iii) of fibroblasts, keratinocytes or endothelial cells on a mixture of poly(3-hydroxybutyrate-co-3-hydroxyvalerate and gelatin-methacryloyl) (PHBV-GelMA) patches [113] (Figure 4).

**Figure 5:**
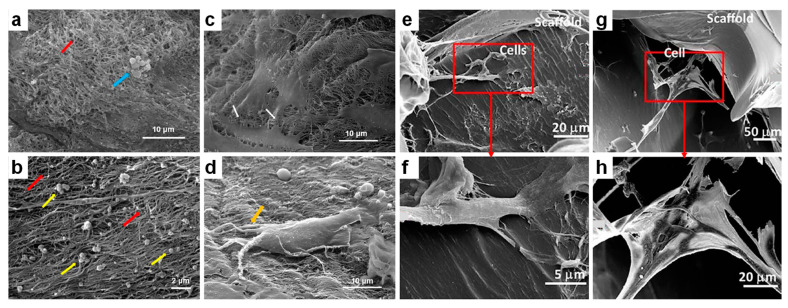
(**a**,**b**) SEM images of CGF scaffolds depicting 3D fibrin networks comprised of interwoven fibers (red arrows), platelets (yellow arrows) and leukocytes (blue arrow). (**c**) SEM image emphasizing the interaction of DPSCs with CGF (white arrows). (**d**) SEM micrograph showing the cellular filopodia (orange arrow) of fully stretched DPSCs on the scaffold. (**e**–**h**) SEM images of scaffold-cells structures at two days culture depicting the morphology and adhesion of bone marrow human MSCs in gelatin-based hydrogels containing about 8% (**e**,**f**) and almost 15% chitosan (**g**,**h**). Images (**f**,**h**) represent the zoom-in of rectangular shapes visible in (**e**,**g**), respectively. Adapted with permission from ref. [127] (**a**–**d**) and ref. [128] (**e**–**h**).

Scanning electron microscopy is a technique that uses a focused beam of electrons to scan a desired sample while detecting the emitted secondary electrons. Characteristics of various types of materials can be identified, including but not limited to surface relief microstructures developed on chitin-derived biopolymers [114], the surface morphology of porous biopolymer nanofibers [107], rough nanostructures [110], as well as many other (biodegradable) structures [20,21,52,111,115,116,117,118,119,120,121]. With SEM, specimens are observed in a vacuum, therefore the studied biological samples are required to be dry and fixed on a hard substrate. Examples of SEM employment in the study of biological samples and cells include the cell growth of the U-2 OS cell line (immortalized human cell line derived from osteosarcoma cells) [122], fibroblasts [123], and hOB cell lines (human osteoblast-like cell) on regular periodic structures of polyethersulfone (PES) [122] or on micropillars of poly(methyl methacrylate (PMMA) [124]. Moreover, SEM can be employed to study the effect of substrate stiffness or biofunctionalization on proliferation and differentiation of periodontal ligament stem cells [125] and adipose-derived stem cells [126]. Furthermore, SEM can be successfully utilized in in vitro studies that aim to reveal the effects of concentrated growth factor (CGF) on cytocompatibility, proliferation and the differentiation of human cells such as dental pulp stem cells (DPSCs; Figure 5a–d) [127] or to evaluate the proliferation and osteogenic differentiation of human MSCs within 3D gelatin-chitosan hydrogels and in the presence of human platelet lysate (Figure 5e–h) [128].

## 5. Insight into the Role of the Surface Topography on Cell Proliferation and Differentiation

Various cellular behaviors such as adhesion, migration, differentiation, cytoskeleton distortion or even gene expression were shown earlier to clearly depend on the changes in surface topography [129]. Therefore, in the final part of this work we aim to emphasize the most recent studies reporting on the relationship between various surface relief patterns based on biopolymers and the proliferation and differentiation of cells to better understand how the two cellular responses are affected by the substrate topography.

### 5.1. The Impact of the Surface Topography on Cell Proliferation

**Figure 6:**
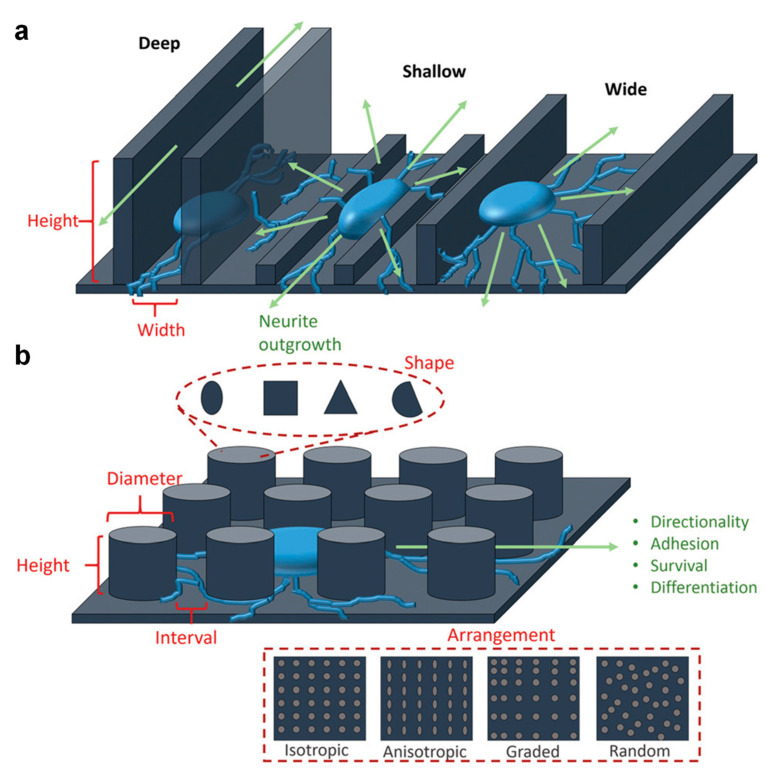
Schematics depicting various parameters that can be controlled within specific topographies and the resulting effects on neural cells: (**a**) continuous topographies represented by grooves of various dimensions are able to affect the direction of cell outgrowth and polarity; (**b**) discontinuous topographies such as pillars of different shape, size and arrangements can alter the adhesion, survival and differentiation of neural cells. Adapted with permission from ref. [129].

There are many parameters related to the topography of a substrate (e.g., the shape and dimension of the surface relief patterns, their periodicity or discontinuity, their arrangements, etc.(see Figure 6) that can be controlled in order to induce various, often highly desired effects, including cell directionality or alignment, and to “force” cells to take a specific shape and to adhere more or less prominently to signal various pathways, to migrate and regenerate [129], etc. As will be further described below, the surfaces used in experiments are displaying surface relief patterns that are either coated with biopolymers [130,131,132,133] or are entirely sculptured in biopolymers [61,134,135,136].

When learning about cellular behaviors (adhesion, proliferation, orientation, etc.), it seems like a good strategy to consider the report of Nagata et al. from the 1990s which was focused on analyzing the directionality of neuroblasts cultured on artificial microstructures [137]. Specifically, the study outlined that the nervous system’s cells are puzzling when trying to control their behavior by changing the classic properties of culture surfaces (for instance, neurons need good adhesion properties with respect to the substrate to proliferate efficiently [138]). Following this direction, Rangappa et al. Performed one of the first experiments in the nervous system area by culturing dorsal root ganglion (DRG) on laminin-coated poly(L-lactide) (PLLA) filaments. Experiments showed that the grown neurites were longitudinally oriented [130]. Moreover, the laminin coating determined an increase in the longitudinal dimension of neurites from 2 mm on an uncoated surface to 5.8 mm on laminin coated filaments [130]. Changes in orientation were further observed in Schwann cells grown on laminin coated poly (methyl methacrylate) (PMMA) films sculptured with multi-width lines patterns [139]. Here, the laminin coating increased the adhesion properties which contributed to a higher proliferation rate and could even lead to the formation of a monolayer of cells that covered the entire substrate surface, with cells displaying a high order or orientation. Schwann cells along with neurons from the DRG explant were further studied by Miller et al. on laminin coated poly (D,L-lactic acid) (PDLA) substrates displaying a groove-like surface relief pattern, the latter having the role of providing not only the physical guidance but also favoring the growth of axons [140]. The laminin coating was also used to cover the filament membranes of poly (acrylonitrile-co-vinyl chloride) (PAN-PVC) along with fibronectin. Both coatings determined the same cell behavior when different diameter fibers were used as the substrate on which neurons and Schwann cells originated from dorsal root ganglion of mice were deposited. When compared to uncoated control fibers, the outgrowth of neurites was increased, and the increase was more pronounced along the laminin and fibronectin coated fibers of subcellular size (5 µm diameter filament bundles) and along the laminin coated fibers of cellular size (30 µm diameter filament bundles) than along the fibers of supracellular size [141].

**Figure 7:**
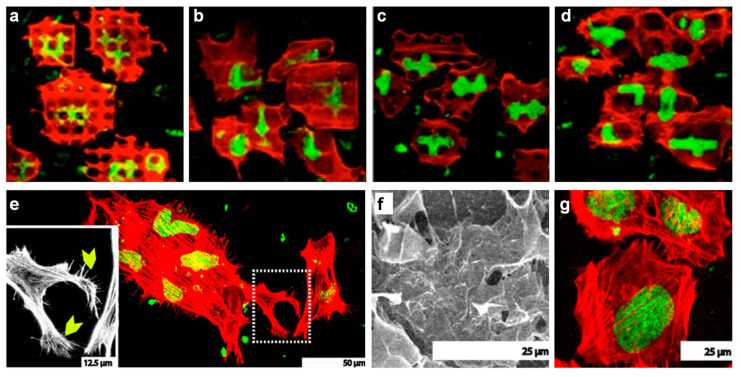
(**a**–**d**) Confocal laser scanning micrographs depicting the behavior of Saos-2 cells on collagen on 8 µm high pillars of different lateral dimensions: 8 × 8 µm^2^ and 4 µm spacing (**a**), 16 × 16 µm^2^ and 4 µm spacing (**b**), 8 × 8 µm^2^ and 8 µm spacing (**c**), 16 × 16 µm^2^ and 8 µm spacing (**d**). (**e**) SEM and confocal (inset) micrographs of the Saos-2 cells cultured on the surfaces patterned with 8 × 8 µm^2^ and 16 × 16 µm^2^ collagen pillars (all spaced at 8 µm), respectively. Images showed that cells had coarse fibers stretching along the cell axis, and distinct, fine filopodia (indicated by the yellow chevrons point to filopodia). (**f**,**g**) SEM (**f**) and confocal (**g**) micrographs of the Saos-2 cells cultured on the plain collagen. Adapted with permission from ref. [133].

Another type of coating frequently used in the cell proliferation research is the collagen type I. Hsu et al. used silicone as a substrate for a Schwann cells culture that, prior to coating with collagen type I, was micropatterned with different models. The results showed that the grooves with larger width/spacing (20/20 µm) and depth led to a more evident increase of the percentage of aligned cells than any other grooves of smaller width/spacing (10/10 µm) and depth [142] (the different behavior of cells depending on the dimensions and types of grooves is schematically represented in Figure 6 [129]). Nonetheless, the alignment of cells on the laminin coated grooves was shown to increase to 60%, as compared to a 51% increase on the collagen type I coated grooves and to only a 41% increase on the uncoated surface [142]. These results indicated that laminin was better favoring the proliferation process than collagen type I. Other studies showed that there is a difference in the adhesion rate of Schwann cells on materials coated with laminin, collagen type I and fibronectin [143], hence the proliferation on collagen type I seems indeed to be weaker than on laminin [142,143] or fibronectin [143]. Even so, collagen type I is over excelling when compared to polymers such as poly (lactic acid-co-glycolic acid) (PLGA). For example, in the bone-area research, osteosarcoma cells (human Saos-2 cell line) grown on collagen and PLGA, both on pillar-like patterns and on flat surfaces, have shown that cells spread more on a collagen surface than on a PLGA surface, demonstrating the superior cell adhesive property of collagen [133]. Moreover, confocal laser scanning microscopy studies further showed that Saos-2 cell proliferation was sensitive to the type of collagen coated micropillars (Figure 7a–d), with the actin fibers being stretched along the cell axis and fine filopodia, and with the abundance of filopodia specifically observed on the patterned surfaces (Figure 7e–g).

Other nervous cells include hippocampal neurons. These can be cultured on polymer substrates previously coated with two biopolymeric systems such as polylysine and laminin (this double coating is being used for enhancing the adhesion of the cells to the substrates) [144]. Various isotropic patterns defined by their symmetry along both axes (dots, squares, grids) and anisotropic patterns such as gratings, triangles and others were designed through soft lithography. These patterns displayed diverse dimensions (with their width, diameter or space between pattern units ranging between 2 and 20 µm) and were used to analyze their specific effects on cells when in contact. For example, the axons grew the longest on the gratings having a width of 5 or 15 µm, compared to the samples exhibiting the narrowest widths with constant interspacing of 2 µm. Compared with axonal growth on planar surfaces, the grating patterns showed an average of 60% greater growth. This percentage was of almost 48% for circular patterns. Moreover, the strongest axon guided growth was seen on gratings and circles compared to any other type of patterns, although the axon branching was very reduced [144].

Bacterial cellulose (BC), a biopolymer used in biomedical, food or chemical products industries, can be produced, purified [145] and further coated with gelatin in order to provide suitable surfaces for human dermal fibroblasts (HDF) cultures (note here that the uncoated BC already determines a higher proliferation rate due to its high tensile strength and a degree of polymerization higher than that of usual cellulose; coating BC with gelatin is further favoring cell interactions [146]). Moreover, such gelatin coated surfaces can be further patterned with 1 µm deep grooves exhibiting different widths (2, 10 and 100 µm). Systematic cell studies revealed that cell migration velocity on substrates with narrower patterns was significantly reduced as compared to flat surfaces, while the cell alignment on grooves exhibiting sizes comparable to the size of the cells was more prominent [146]. Moreover, an in vivo experiment showed that the groove patterns on a BC based skin wound dressing were able to favor the infiltration of fibroblasts and deposition of collagen necessary for wound healing.

In vivo, macrophages and fibroblasts have the tendency to adhere to implanted materials and to widely spread, leading to complications [147]. Considering this, there is a need to find a way to develop antiadhesive topographies for improving their utility in implant engineering. Antiadhesive properties of surfaces exhibiting relief patterns are based on the physical interference of the topographic features with the determination and maturation of focal adhesion. In order to study the behaviors of the HDF and macrophages on antiadhesive substrates covered with various patterns, the former were cultured on biocellulose and on polydimethylsiloxane (PDMS) substrates, both coated with fibronectin. These substrates were then imprinted with patterns such as hexagonal pits with different diameters (6–20 µm), lateral spacings (6–23 µm distance between pits) and shapes (perfectly isotropic centered-hexagonal and quasi-isotropic squares) [148]. The adhesion rate-based results showed that, when compared with flat surfaces, the spread of HDF cultured for 72 h on fibronectin/PDMS and fibronectin/biocellulose decreased with almost 60% on almost all patterned surfaces, the only exceptions being represented by the hexagonal or square arrays with a diameter of 10 µm and a lateral spacing of 13 µm. The highest reduction of adhesion rate (65%) was seen on hexagonal and square pits having a diameter of 5 µm and a lateral spacing of 10 µm. Interestingly, after one week of cell culture, the adhesion decreases further, finally reaching 75% [148]. Moreover, the long-term interaction between HDF and the antiadhesion biocellulose-based topographies decreased the proliferation rate to only 10%. Furthermore, a detailed analysis of the circularity of the cells revealed that the biocellulose pits did not sustain the elongation of the cells seen on PDMS substrates, indicating that biocellulose-based geometries reduced the interactions between the substrate and the cells [148].

A slightly different approach to study the process of cell proliferation is to replace the surface relief patterns coated with biopolymers with surface relief patterns entirely sculptured within biopolymers. For instance, collagen can be used to fabricate films that can further undergo patterning [149]. Specific patterns can also be obtained from chitosan [61], fibronectin, cellulose, various proteins, enzymes, etc. (see Table 1). Additionally, collagen may be combined with other materials such as glycosaminoglycan to get scaffolds for tissue engineering [150]. This variety of surfaces has its own use as there are always advantages and disadvantages to using them in cell studies. For example, in films prepared from collagen extracted from rat′s tails, the adhesion of cells can be weak and, therefore, osteoblasts may not grow as expected [149]. For adhesion, osteoblasts prefer fibronectin to the detriment of type I and IV collagen. Furthermore, while the adhesion is weaker on laminin and type V collagen, osteoblasts do not adhere to type III collagen [151]. These observations are highly important when dealing with tissue engineering applications based on Schwann cells and neurons [141,142,152,153,154], osteoblasts [149], fibroblasts [155], or human corneal keratocytes and retinal pigment epithelial cells [156] (see Table 2).

Vrana et al. studied the alteration of properties of collagen-based micropatterned films by growing keratocytes and epithelial cells with the goal to eventually design a functional artificial cornea [134]. Here, while the unseeded collagen films suffered a reduction of strength, the growth of keratocytes improved the mechanical behavior of the films. On the other hand, the pigment epithelial cell line D407 seeded on the same films deteriorated the mechanical properties of the latter. Instead, collagen-patterned ridges oriented keratocytes and gave them an elongated shape. Moreover, after a period of three weeks this behavior changed, as the keratocytes had the ability to adhere to the inclined walls of the ridges once they occupied the base of the patterns [134]. Furthermore, relief patterns changed the cytoskeletal arrangement of keratocytes, the f-actin filaments being aligned with the groove direction after a period of seven days. Nonetheless, when considering the proliferative rates, the authors have shown that D407 cells grew better on flat surfaces, as patterns prevented the formation of cell-to-cell contacts. Instead, keratocytes better conformed to relief patterns and led to an oriented layer of cells once the relief patterns were degraded by the enzymes produced by keratocytes. The resulting films seeded with keratocytes presented better mechanical properties as the proliferation of cells compensated for the loss of the substrate integrity [134]. A relation between the cell proliferation and the presence of relief patterns was further demonstrated when investigating the role played by the size of collagen-glycosaminoglycans pores on the growing of osteoblasts. The results showed that after a period of seven days, osteoblasts proliferated with a higher rate in larger-sized pores made in collagen-based scaffolds, as such a size of the pores better favored the migration of the cells [150].

**Figure 8:**
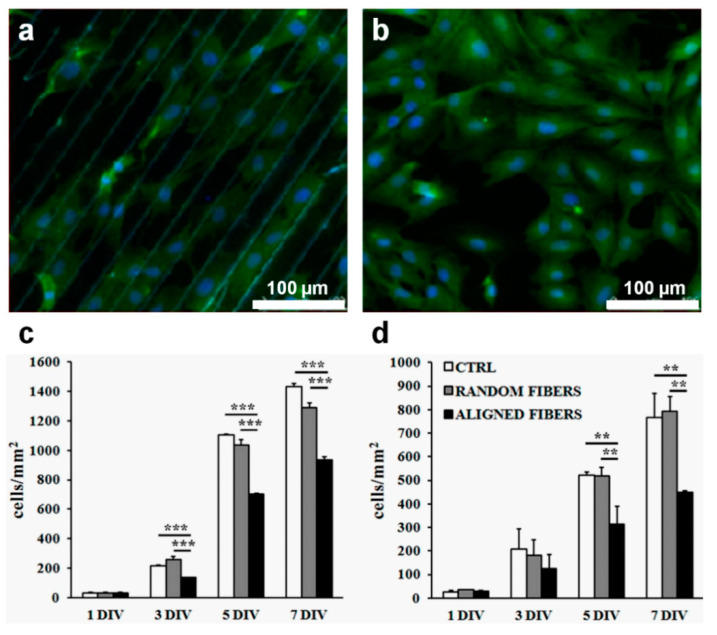
(**a**,**b**) Optical micrographs revealing the behavior of Schwann cells on a substrate patterned with 30 µm wide chitosan grooves (**a**) and on a flat chitosan control substrate (**b**). (**c**,**d**) Proliferation of RT4-D6P2T (**c**) and primary Schwann cells (**d**) on substrates covered with aligned and random gelatin fibers, as well as on control poly-L-lysine coated coverslips. Asterisks ** and *** indicated significant statistical differences with *p* ≤ 0.01 and *p* ≤ 0.001, respectively. Adapted with permission from ref. [61] (**a**,**b**) and ref. [154] (**c**,**d**).

More recent studies placed neurons or Schwann cells on patterned chitosan surfaces [61] and on gelatin electro spun fibrous substrates [154]. On chitosan ridge/groove patterns, Schwann cells exhibited an orientational growth, as such patterns controlled the alignment of cell growth (Figure 8a). This was not the case for the flat chitosan surfaces where cells grew isotropically (Figure 8b) [61]. On the other hand, analyzing the effect of the orientation of gelatin fibers on primary Schwann cells and the RT4-D6P2T Schwann cells line, Gnavi et al. have demonstrated that although the alignment of fibers actually reduced the adhesion and proliferation rates as compared to random fibers (Figure 8c,d), it still favored the alignment of actin filaments of Schwann cells [154]. The reported data showed that Schwann cells were elongated with their longitudinal body along the aligned gelatin fibers. Similarly, when cultured on gelatin aligned fibers, the B5011 neuron cells were aligned and exhibited parallel axon growth. The authors concluded that the orientation of fibers could be used to modulate Schwann cells and axon organization in vitro [154]. Other interesting examples and aspects of the cell proliferation-biosurface relief pattern relation can be inferred from the data briefly described in Table 2.

### 5.2. The Impact of the Surface Topography on Cell Differentiation

As we mentioned above, the culture medium may contain different chemical stimuli for guiding cells to differentiate into a specific type of cell. Moreover, the process of cell differentiation can be also initiated by different biomaterials having specific topographies [157]. For example, the most common stem cells used in the differentiation studies on substrates with various topographical features are MSCs [158,159,160,161,162] and iPSCs [163]. Therefore, hereafter we analyze the cell differentiation behavior with respect to specific surface relief patterns that were either coated with biopolymers or directly sculptured in biopolymer films [157,160].

Following the idea that many tissues have an anisotropic architecture, Lanfer et al. created aligned structures out of collagen type I using a microfluidic set-up. Furthermore, they incorporated glycosaminoglycan heparin for studying multiple extracellular matrix (ECM) components at once, and researched multiple differentiation lineages of MSCs (osteogenic, adipogenic, chondrogenic) [158]. The most important impact of the substrate was observed in the osteogenic differentiation, where the osteogenic medium induced the formation of mineralized nodules on substrates of aligned collagen structures. In comparison, these structures were missing on flat glass substrates [158]. Moreover, the substrate comprised of aligned collagen fibers influenced the osteogenesis of human MSCs by directing the ordered matrix mineralization. The ability of undifferentiated stem cells to sense the mechanical properties of their growing environment and to differentiate accordingly was further demonstrated by Park et al. [164]. They have shown that bone marrow MSCs, which have the ability to differentiate into smooth muscle cells or chondrogenic cells, can become either one of these types of cells, depending on the stiffness of the matrix there are growing on. MSCs on soft collagen substrates spread less and showed lower proliferation rate than MSCs cultured on stiff collagen substrates. Also, the stiff matrix promoted the differentiation into smooth muscle cells while the soft matrix promoted the differentiation into chondrogenic and adipogenic cell lines [8].

A new approach to test the relationship between substrate topography and cell differentiation process was developed in 2014 by Younesi et al., who have built a 3D scaffold of anisotropically oriented collagen fibers (collagen was used for its properties that support the attachment and growth of some primary cells). This structure mimicked the properties of a native tendon presenting a porosity of 80%. MSCs cultured on the scaffold faced tenogenic differentiation without the presence of the growth factors. The differentiation was seen by the presence of tenomodulin, cartilage oligomeric matrix protein (COMP) and collagen type I in the synthetized matrix (specific tenogenic markers that were up-regulated), and represents a promising alternative for repairing tendons and ligaments [160].

Another biopolymer that can be used in bio-coating to improve seeding surfaces is fibronectin [159,161,165]. For instance, PLGA coated with fibronectin can be patterned with spatially defined geometries and further used for establishing control over the morphology of bone marrow derived human MSCs and eventually for altering the cell differentiation [159]. The results showed that while cells that have been grown on 20 µm wide strips were highly elongated and exhibited an area coverage of ~2000 µm^2^, cells grown on flat, unpatterned surfaces displayed a much larger area coverage of ~ µm^2^. Moreover, an ulterior analysis on gene expression indicated that while the elongated cells exhibited up-regulation of several markers associated with neurogenesis and myogenesis, the markers associated with osteogenesis were down-regulated or remained at its nominal level. This demonstrated that the mechanical deformation of cells can be translated into biochemical response and suggested the idea that cell differentiation can be altered by the substrate, in absence of other differentiation factors [159].

As technology is advancing, new ways of improving the materials’ properties are discovered. Substrates coated with rod-shaped turnip mosaic virus (TMV) and spherical turnip yellow mosaic virus (TYMV) were used for culturing bone marrow derived MSCs. This represents a new approach in studying osteogenic differentiation. By analyzing osteogenic markers such as bone morphogenetic protein 2 (BMP-2), an acceleration of osteogenic differentiation process by seven days in both cases was shown [166]. An exact explanation for these mechanisms is not yet available, but some studies showed that cells may use some molecular mechanisms for sensing different topographies which are able to coordinate the focal adhesion of cells [34].

Also, a small guanosine triphosphatase (GTPase) named RhoA and Rho-associated kinase (ROCK) are known to have effects on the control of cell fate by cell spreading through their action on actomyosin contractility [167]. Other studies suggest that some sort of cell membrane and cytoskeleton stress induce cytoskeletal tension mediated by the RhoA/ROCK complex, resulting in the beginning of osteogenesis. In this way, it can be supposed that the virus nanoparticles supply some topographical features that induce mechanical stress to the cell membrane which enables it to trigger an earlier osteogenic process [168].

Furthermore, for the onset of fibrosis, the cells not only have to adhere and to proliferate, but they also have to differentiate for the deposition and contraction of the fibrotic matrix on the surface of implants. To study the differentiation of fibroblasts into contractile myofibroblasts, the expression of α-Smooth Muscle Actin (α-SMA) of cells on different dimension pits, on substrates covered with both hexagonal and square patterns, was analyzed [148]. The most important reduction of α-SMA expression was encountered on the hexagonal disposition of pits with a diameter of 5 µm and a lateral spacing of 10 µm. When compared to the cells cultured on TCP (tissue culture plastic) or MED6015 (medical grade silicone), results have shown that the biomaterial properties did not support the activation of the required signals for differentiation, and that the upgrade of the biocellulose surface with pits-patterns introduced a significant additional inhibition of differentiation, preventing the fibrosis [148].

**Figure 9:**
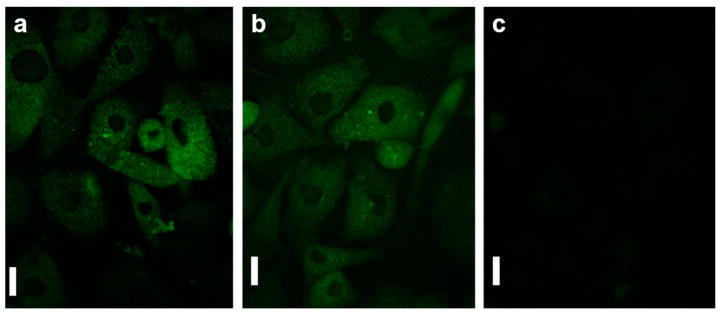
Fluorescence micrographs depicting the staining of OCN cells cultured on substrates patterned with 4 µm sized tMPs (**a**), on substrates patterned with 2 µm sized tMPs (**b**) and on flat surfaces (**c**), respectively. Scale bars represent 15 μm. Adapted with permission from ref. [161].

An interesting and innovative approach proposed the use of fibronectin coating on a different substrate. Starting from the fact that cellular functions such as proliferation and differentiation can be upregulated by enhancing the focal adhesion (FA) between cells, ECM and intracellular actin polymerization (AP), Seo et al. improved the culture substrate by patterning it with tailor-made micrometer-sized pits (tMP) coated with fibronectin [161]. Mouse MSCs (C3H10T1/2) were cultured on tMPs substrates and the obtained results showed that although the cell spreading area was not affected by this topography, the cells FAs were increased together with AP and traction forces. Therefore, the osteogenic differentiation increased as well. This was observed by reverse transcription polymerase chain reaction (RT-PCR) techniques and western blotting that showed upregulation of specific osteogenic markers such as alkaline phosphatase (ALP), collagen type I, osteocalcin (OCN) and runt-related transcription factor 2 (Run × 2)/core-binding factor 1 (Cbfa1). OCN is a late dominant marker of osteogenic differentiation and, as it is emphasized in Figure 9, its intensity was significantly higher in cells grown on the tMP surfaces than those on the flat surfaces [161].

**Figure 10:**
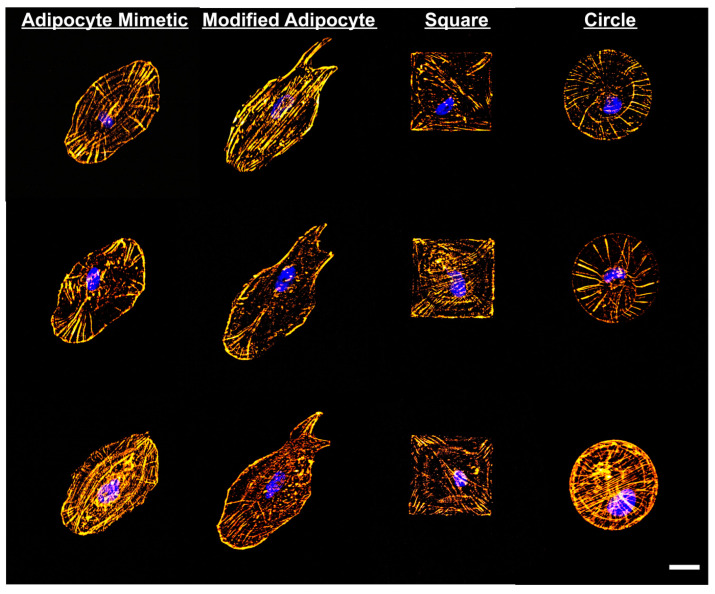
Actin cytoskeleton of human stem cells on adipocyte mimetic fibronectin patterns, modified adipocyte fibronectin patterns as well as square and circle fibronectin patterns. Here gold color stands for F-actin while blue color stands for the nucleus. The scale bar is 25 μm. Reproduced with permission from ref. [165].

Fibronectin was further employed by Shukla et al., who fabricated micropatterned fibronectin arrays of biomimetic geometries that replicated the morphology aspect of mature cells. Adipocytes cultured in 2D were imaged and used to create biomimetic virtual masks which were then employed to pattern the fibronectin surfaces via the laser scanning lithography. Reported results pointed out a clear influence of the pattern geometry on human MSC differentiation (Figure 10). While human MSCs seeded on nonpatterned fibronectin surfaces in differentiation medium showed positive staining for both lipids and ALP (~10% of the cells stained positive for lipids, ~20% of the cells stained positive for ALP and the remaining cells were not positive for either marker), the MSCs cultured on mimetic patterns in the same differentiation medium showed positive staining for lipids (45% of the cells) and ALP staining was not present in any of the MSCs [165]. In conclusion, the mimetic geometry determined the human MSCs to differentiate into adipocytes.

For culturing of human MSCs cells, fibronectin can be also used in combination with gelatin to coat the PDMS substrates. The latter can be patterned with 10 μm or 20 μm wide and 3 μm deep grooves (the depth of grooves was chosen to match the few micrometers sized heart muscle matrix fibers) [169]. After the visualization of the arranged cytoskeleton, the changed morphology of the cells, as well as the enhanced expression of GATA4, troponin I and troponin T on the surface covered with relief patterns, it was concluded that the 20 μm wide grooves promoted better the cardiomyogenic differentiation of human MSCs [169]. These results were of significant impact, knowing that the arrangement of the cytoskeleton, especially of actin filaments, is important in a lot of signaling pathways [168].

Another interesting study was implemented using scaffolds based on loosely packed silk nanofibers on which the MC3T3-E1 pre-osteoblast mice cell line was cultured. The physicochemical properties of the cells grown on the aligned fibers were compared to the properties of the cells cultured on random-structured fibers. Results showed that cells on the aligned structures not only exhibited an elongated morphology and a more ordered arrangement, but also presented faster and deeper infiltration. The latter promoted proliferation and osteogenic differentiation of the pre-osteoblasts, as indicated by the expression of ALP activity and the presence of the macroscopic mineral nodes after 14 days [170]. Note that the importance of physicochemical properties of the substrates on cell differentiation is highly important, especially when a controlled differentiation of cells is desired. This was exemplified inclusively in a 3D extracellular matrix, when growing human amniotic mesenchymal stem cells (AMSCs) on fibrin hydrogels loaded or not with gold nanowire, and exhibiting different elasticity of the substrate. The latter was shown to clearly affect the osteogenic or chondrogenic differentiation [162].

Besides MSCs, iPSCs are also used for studying differentiation properties on surface-modified substrates. For instance, aligned chitosan fibers can mimic the native tendon’s microstructure and its mechanical properties. Therefore, while human iPSCs could differentiate into MSCs on a smooth surface (with the differentiation process being confirmed by the presence of characteristic MSC surface markers), iPSCs subsequently cultured on well aligned fibers differentiated into tenocyte-like cells through the activation of the mechanic-signal pathway [163]. Further examples that describe the direct linking of the surface relief patterns to the cell differentiation process are enlisted in Table 2.

**Table 2:** Characteristics of several topographies that impact cell behavior.

Surface Patterns	Material	Cell Type	Results	Ref.
Proliferation				
Filaments	PLLA/laminin	Rat Schwann cells and neurons from DRG	Neuron’s neurites grown on the coated filaments were longitudinally oriented and were longer than those uncoated	[130]
Ridge Depth: 30 µm Top width: 10 µm Base width: 2 µm	Collagen	Human corneal keratocytes and retinal pigment epithelial cells (D407 line)	Patterns changed the cytoskeletal arrangement of keratocytes, f-actin filaments being aligned longitudinally; D407 cells grow better on flat surfaces	[134]
Lines Width: 10–50 µm	PMMA/laminin	Rat Schwann cells	The lines oriented the cells longitudinally (smaller lines increased the orientation degree); laminin-patterned areas increased the cells adhesion, thus induced proliferation	[139]
Grooves Width: 10 µm	PDLA/laminin	Rat Schwann cells and neurons from sciatic DRG	Grooves provided physical guidance and laminin assured stronger adhesion that promotes proliferation	[140]
Filaments Diameter: 5 µm; 30 µm; 100 µm; 200 µm; 500 µm	PAN-PVC/laminin PAN-PVC/fibronectin	Rat Schwann cells and neurons from DRG	Alignment and outgrowth of neurites were most prominent on filament bundles with individual filament diameters between 5 and 30 µm for laminin and 5 µm for fibronectin	[141]
Grooves Width: 10/20 µm Spacing: 10/20 µm Depth: 0.5/1.5/3 µm	Silicon/laminin and sillicon/collagen type I	Rat Schwann cells	20/20/1.5 µm grooves had the biggest impact (cells alignment). Laminin coated grooves increased the alignment and adhesion of 60% of the cells; the collagen type I coating increased the alignment and adhesion of the 51% of the cells compared to uncoated substrates	[142]
Anisotropic (gratings) and isotropic (dots, grids) patterns	PDMS/polylysine and laminin coating	Hippocampal neurons	Gratings promoted directional axonal growth and most enhanced axonal outgrowth	[144]
Grooves Width: 2 µm (P2); 10 (P10); 100 µm (P100)	PDMS/biocellulose coating	Fibroblasts	P2 and P10 grooves showed reduced migration of cells; grooves with size closer to the cell size had stronger alignment effects	[146]
Pores Diameter: 85–325 µm	Collagen-glycosaminoglycan	MC3T3-E1 pre-osteoblast cell line	The highest cell proliferation rate was on largest pore size	[150]
Proliferation and differentiation				
Pits Diameter: 6–20 µm Spacing: 6–23 µm	PDMS/biocellulose coating	Human dermal fibroblasts and macrophages	The most enhanced reduction of adhesion rate was given by pits with diameter of 5 µm and 10 µm distance between pits. Also, pits with same sizes showed the higher reduction of cell differentiation	[148]
Differentiation				
Aligned fibers	Collagen I and heparin	Human MSCs; C2C12 myoblasts cell line	The alignment of the collagen fibers and the addition of heparin didn’t have any effect on the adipogenic differentiation of MSCs; instead, the aligned fibers promoted skeletal muscle morphogenesis	[158]
Grooves Width: 10/10 µm; 20/20 µm; 30/30 µm; 50/50 µm	Chitosan	Schwann cells from lumbar dorsal root and sciatic nerves of rats	Schwann cells on 30/30 µm patterns kept orientationally growth and increased proliferation compared to cells seeded on the other patterns size	[61]
Spots Diameter: 500 µm	Fibronectin and collagen I (mixture) coating (+ growth factors)	Mouse embryonic stem cells	Stem cells cultured on substrate spots having hepatocyte growth factor exhibited hepatic differentiation and loss of pluripotency; co-culture with non-parenchymal liver cells enhanced the differentiation rate	[171]
Micropits Area: 3 × 3 µm^2^ Height: 2 or 4 µm	Fibronectin coating	C3H10T1/2 mouse MSCs line	4 µm height micropits induced acceleration of osteogenic differentiation	[161]
Gold nanowires (AuNWs) based structures	Fibrin hydrogel/ AuNWs	Human amniotic MSCs	AuNWs in stiff substrate promoted osteogenic differentiation and AuNWs in soft substrate promoted chondrogenic differentiation	[162]
Anisotropically aligned fibers Diameter: ~891 nm	Chitosan	Human-induced pluripotent stem cells	Tenogenic differentiation through activating mechanic-signal pathway	[163]
Biomimetic geometry	Fibronectin coating	Human MSCs	Adipocyte mimetic geometries showed increased MSCs adipogenesis properties	[165]
Grooves: Width: 10/20 µm Spacing: 20 µm Depth: 3 µm	PDMS/fibronectin and gelatin coating	Human MSCs	20 µm width grooves accelerated osteogenic differentiation	[169]
Aligned/fibrous scaffolds	Silk fibroin	MC3T3-E1 pre-osteoblasts	Aligned scaffold promoted cell proliferation and osteogenic differentiation	[170]

## 6. Outlook and Future Perspectives

In this review we emphasize that various surface relief patterns of specific dimension and function can have a significant impact on many aspects of cell behavior and development. Firstly, coating the usual substrates made by common materials such as glass or PDMS with biopolymers, mostly enhances the adhesion of cells. Biopolymers that help increase the adhesion properties of the cells are mainly the adhesion proteins that can be found in the ECM, such as collagen, fibronectin, laminin or fibronectin. As many studies show, patterns that can increase the cell adhesion are those having sizes close to the cell size that permit the cell to spread entirely on the pattern surface. The mechanisms by which cells recognize the limits of their environment, in this case, the limits of the pattern they grow on, are still rather unknown and many studies have to be developed for explaining the phenomenon. Relief patterns such as grooves of chitosan can elongate the cells, and for neurons, they can promote the axon guidance or outgrowth of dendrites. Also, relief patterns can promote the aligned growth of cells by downregulating the expression levels of N-cadherin. Moreover, the patterns functionalized with collagen can decrease the deformation of the nucleus, this effect being clearly visible when comparing it on other substrates such as the stiff PLGA. Furthermore, analyzing the properties of the cells such as adhesion, migration, elongation, and growth of the neural extensions gives valuable information related to the changes in cell proliferation. For example, cells like Schwann cells that need to strongly adhere to a substrate for proliferation, will certainly increase spreading and growing once that adhesion rate is enhanced.

We also highlight that the relief patterns with different dimensions sculptured in biopolymer films have an impact on the differentiation of the cells via some genetic expression changing and signaling pathways such as BMP pathway that intervene in osteogenic differentiation. Once the cell is spreading and some contractile tension appears, the signaling pathway determines the differentiation line. Of course, these processes are very complex and more and more signaling pathways activate one after the other. The substrate stiffness can drive the differentiation to one direction or another, the same as other growth factors in the culture, or other cells being present as co-culture, etc. Therefore, we can conclude that the process of culturing different types of cells on various types of substrates (coated or integrally made of biopolymers) exhibiting surface relief patterns of specific shapes and dimensions can tailor the evolution of cells. Mechanisms that stay behind all of the cellular changes are very complex and strongly depend on the chemical and physical properties of the materials the cells grow on. Moreover, the most important observation that can be drawn is the fact that by developing functionalized patterned materials made of biopolymers, the bone regeneration can be accelerated, the implants can be easier accepted by the host organism and the acquisition of specialized cells can be facilitated by controlling the cell differentiation faith.

Lastly, there is a question of how to translate pattern-based materials from preclinical models to clinical use. The starting point, and the basis for regulatory Food and Drug Administration (FDA) approval, is to control the design of such platforms, taking into consideration the heterogeneity of most of the biopolymer-based approaches, the variation, anisotropy, material voids and other challenges encountered in the fabrication process [172]. A validation from the experiment to the industry might be achieved by involving new technologies, such as 3D printing, which could provide the means for the large-scale use and essentially contribute to the improvement of reproducibility and quality of the final products [173]. Moreover, by using 3D printing, the customization of the device can be rapidly achieved in an economically feasible manner, which is not a negligible factor considering the high costs imposed by the long-term treatments such as those used for bone regeneration, wound healing or the general recovery of the patient [174]. It is also likely to facilitate and better interface the manufacturing sector with the clinical one with regard to the testing and validation of such products, which is still a critical point for future translation. Therefore, adding to such novel technologies new patterned materials as are those provided by nanolithography, with obvious advantages over their microscale counterparts and capability to influence biology at the nanoscale, seems to be a rational approach that might improve the way forward to the clinic.

## Figures and Tables

**Table 1:** Summary of various biopolymeric surface relief patterns that can be created using top-down and bottom-up lithographic methodologies.

Lithography	Patterned Material	Resulting Pattern	Pattern Dimension	Ref.
DLW	chitosan, starch	pores	μm size	[13]
UV light	silk protein	non-spherical particles	several μm	[18]
UV light	wool keratin protein	lines circular patterns crosses triangles	2 μm/width 3 μm/diameter 3 μm/width tens of μm	[19]
EBL	sugar-based polymer	moth-eye patterns	120 nm/period	[43]
EBL	biotinylated PEG	pads	~10 μm	[5]
IBL	DNA oligonucleotides neutravidin anti-mouse IgG	line assays complex stripes-based patterns	1–2 μm/width down to 100 nm/width	[57]
NIL	chitosan	lines circular pillars	10 μm/width 500 nm/diameter	[61] [29]
NIL	proteins	lines	700 nm/period	[58]
NIL	gelatins/genipin	grooves holes pillars	500 nm/width 500 nm/diameter 100 nm/diameter	[22]
NIL	cellulose	holes lines square pillars rhombus pillars holes	400 nm/diameter 140 nm/width 1 μm/diameter 600 nm/width 600 nm/diameter	[28] [24]
μCP	protein/Sylgard 527	arrays of nanodots	200 nm × 200 nm	[62]
μCP	biomolecules/poly(4-aminostyrene)	stripes pads	~2 μm/width ~7 μm/diameter	[27]
μCP	silk	lines	hundreds of μm/width	[25]
μCP	neutravidin/biotin	arrays of nanodots	~62 nm/diameter	[63]
μCP	amyloid	spider web arrays	hundreds of μm/width	[61]
TCSPL	enzyme	rectangles squares lines dots	4.5 μm × 1.5 μm 100 nm ×100 nm 8–9 nm/width 8 nm/diameter	[23]
PL	streptavidin	patches	15 nm/diameter	[66]
DNSA	DNA	squares disks five-point stars rectangles triangles	~100 nm/diameter ~100 nm/diameter ~100 nm/diameter ~100 nm/diameter ~100 nm/diameter	[26]

## Data Availability

Not applicable.

## References

[1] Saiz N., Mora-Bitria L., Rahman S., George H., Herder J.P., Garcia-Ojalvo J., Hadjantonakis A.-K. (2020). Growth-Factor-Mediated Coupling between Lineage Size and Cell Fate Choice Underlies Robustness of Mammalian Development. Elife.

[2] de Souza Santos R. (2018). Sex and Media: Considerations for Cell Culture Studies. ALTEX.

[3] Yue B. (2014). Biology of the Extracellular Matrix: An Overview. J. Glaucoma.

[4] Bhattacharjee P., Cavanagh B.L., Ahearne M. (2020). Effect of Substrate Topography on the Regulation of Human Corneal Stromal Cells. Colloids Surf. B.

[5] Rusen L., Cazan M., Mustaciosu C., Filipescu M., Sandel S., Zamfirescu M., Dinca V., Dinescu M. (2014). Tailored Topography Control of Biopolymer Surfaces by Ultrafast Lasers for Cell–Substrate Studies. Appl. Surf. Sci..

[6] Ross A.M., Jiang Z., Bastmeyer M., Lahann J. (2012). Physical Aspects of Cell Culture Substrates: Topography, Roughness, and Elasticity. Small.

[7] Ferrari M., Cirisano F., Morán M.C. (2019). Mammalian Cell Behavior on Hydrophobic Substrates: Influence of Surface Properties. Colloids Interfaces.

[8] Park J.S., Chu J.S., Tsou A.D., Diop R., Tang Z., Wang A., Li S. (2011). The Effect of Matrix Stiffness on the Differentiation of Mesenchymal Stem Cells in Response to TGF-β. Biomaterials.

[9] Arango M.-T., Quintero-Ronderos P., Castiblanco J., Montoya-Ortíz G. (2013). Cell Culture and Cell Analysis. Autoimmunity: From Bench to Bedside.

[10] Malm M., Saghaleyni R., Lundqvist M., Giudici M., Chotteau V., Field R., Varley P.G., Hatton D., Grassi L., Svensson T. (2020). Evolution from Adherent to Suspension: Systems Biology of HEK293 Cell Line Development. Sci. Rep..

[11] Jensen C., Teng Y. (2020). Is It Time to Start Transitioning From 2D to 3D Cell Culture?. Front. Mol. Biosci..

[12] Nara S., Chameettachal S., Ghosh S. (2014). Precise Patterning of Biopolymers and Cells by Direct Write Technique. Mater. Technol..

[13] Castillejo M., Rebollar E., Oujja M., Sanz M., Selimis A., Sigletou M., Psycharakis S., Ranella A., Fotakis C. (2012). Fabrication of Porous Biopolymer Substrates for Cell Growth by UV Laser: The Role of Pulse Duration. Appl. Surf. Sci..

[14] Christopherson G.T., Song H., Mao H.-Q. (2009). The Influence of Fiber Diameter of Electrospun Substrates on Neural Stem Cell Differentiation and Proliferation. Biomaterials.

[15] Sevcik E.N., Szymanski J.M., Jallerat Q., Feinberg A.W. (2017). Patterning on Topography for Generation of Cell Culture Substrates with Independent Nanoscale Control of Chemical and Topographical Extracellular Matrix Cues. Curr. Protoc. Cell Biol..

[16] Nguyen A.T., Sathe S.R., Yim E.K.F. (2016). From Nano to Micro: Topographical Scale and Its Impact on Cell Adhesion, Morphology and Contact Guidance. J. Phys. Condens. Matter..

[17] Ravi M., Paramesh V., Kaviya S.R., Anuradha E., Solomon F.D.P. (2015). 3D Cell Culture Systems: Advantages and Applications. J. Cell. Physiol..

[18] Pal R.K., Kurland N.E., Jiang C., Kundu S.C., Zhang N., Yadavalli V.K. (2016). Fabrication of Precise Shape-Defined Particles of Silk Proteins Using Photolithography. Eur. Polym. J..

[19] Zhu S., Zeng W., Meng Z., Luo W., Ma L., Li Y., Lin C., Huang Q., Lin Y., Liu X.Y. (2019). Using Wool Keratin as a Basic Resist Material to Fabricate Precise Protein Patterns. Adv. Mater..

[20] Jiang J., Qin N., Tao T.H. (2020). Nanomanufacturing of Biopolymers Using Electron and Ion Beams. J. Micromech. Microeng..

[21] Handrea-Dragan M., Botiz I. (2021). Multifunctional Structured Platforms: From Patterning of Polymer-Based Films to Their Subsequent Filling with Various Nanomaterials. Polymers.

[22] Makita R., Akasaka T., Tamagawa S., Yoshida Y., Miyata S., Miyaji H., Sugaya T. (2018). Preparation of Micro/Nanopatterned Gelatins Crosslinked with Genipin for Biocompatible Dental Implants. Beilstein J. Nanotechnol..

[23] Liu X., Kumar M., Calo A., Albisetti E., Zheng X., Manning K.B., Elacqua E., Weck M., Ulijn R.V., Riedo E. (2019). Sub-10 Nm Resolution Patterning of Pockets for Enzyme Immobilization with Independent Density and Quasi-3D Topography Control. ACS Appl. Mater. Interfaces.

[24] Zhou Y., Li Y., Dundar F., Carter K.R., Watkins J.J. (2018). Fabrication of Patterned Cellulose Film via Solvent-Assisted Soft Nanoimprint Lithography at a Submicron Scale. Cellulose.

[25] Ganesh Kumar B., Melikov R., Mohammadi Aria M., Ural Yalcin A., Begar E., Sadeghi S., Guven K., Nizamoglu S. (2018). Silk-Based Aqueous Microcontact Printing. ACS Biomater. Sci. Eng..

[26] Rothemund P.W.K. (2006). Folding DNA to Create Nanoscale Shapes and Patterns. Nature.

[27] Wang Z., Xia J., Luo S., Zhang P., Xiao Z., Liu T., Guan J. (2014). Versatile Surface Micropatterning and Functionalization Enabled by Microcontact Printing of Poly(4-Aminostyrene). Langmuir.

[28] Espinha A., Dore C., Matricardi C., Alonso M.I., Goñi A.R., Mihi A. (2018). Hydroxypropyl Cellulose Photonic Architectures by Soft Nanoimprinting Lithography. Nat. Photonics.

[29] Heedy S., Pineda J.J., Meli V.S., Wang S.-W., Yee A.F. (2022). Nanopillar Templating Augments the Stiffness and Strength in Biopolymer Films. ACS Nano.

[30] Lawrence B.D., Pan Z., Liu A., Kaplan D.L., Rosenblatt M.I. (2012). Human Corneal Limbal-Epithelial Cell Response to Varying Silk Film Geometric Topography In Vitro. Acta Biomater..

[31] Lynch C.R., Kondiah P.P.D., Choonara Y.E. (2021). Advanced Strategies for Tissue Engineering in Regenerative Medicine: A Biofabrication and Biopolymer Perspective. Molecules.

[32] Qian T., Wang Y. (2010). Micro/Nano-Fabrication Technologies for Cell Biology. Med. Biol. Eng. Comput..

[33] Harawaza K., Cousins B., Roach P., Fernandez A. (2021). Modification of the Surface Nanotopography of Implant Devices: A Translational Perspective. Mater. Today Bio.

[34] Fedele C., Mäntylä E., Belardi B., Hamkins-Indik T., Cavalli S., Netti P.A., Fletcher D.A., Nymark S., Priimagi A., Ihalainen T.O. (2020). Azobenzene-Based Sinusoidal Surface Topography Drives Focal Adhesion Confinement and Guides Collective Migration of Epithelial Cells. Sci. Rep..

[35] De Masi A., Tonazzini I., Masciullo C., Mezzena R., Chiellini F., Puppi D., Cecchini M. (2019). Chitosan Films for Regenerative Medicine: Fabrication Methods and Mechanical Characterization of Nanostructured Chitosan Films. Biophys. Rev..

[36] Scaccini L., Mezzena R., De Masi A., Gagliardi M., Gambarotta G., Cecchini M., Tonazzini I. (2021). Chitosan Micro-Grooved Membranes with Increased Asymmetry for the Improvement of the Schwann Cell Response in Nerve Regeneration. Int. J. Mol. Sci..

[37] Yulianto E., Chatterjee S., Purlys V., Mizeikis V. (2019). Imaging of Latent Three-Dimensional Exposure Patterns Created by Direct Laser Writing in Photoresists. Appl. Surf. Sci..

[38] He W., Poker D.B., Gonsalves K.E., Batina N. (2003). Micro/Nano Machining of Polymeric Substrates by Ion Beam Techniques. Microelectron Eng..

[39] Hwang I.-T., Oh M.-S., Jung C.-H., Choi J.-H. (2014). Direct Patterning of Poly(Acrylic Acid) on Polymer Surfaces by Ion Beam Lithography for the Controlled Adhesion of Mammalian Cells. Biotechnol. Lett..

[40] He C., Feng Z., Shan S., Wang M., Chen X., Zou G. (2020). Highly Enantioselective Photo-Polymerization Enhanced by Chiral Nanoparticles and in Situ Photopatterning of Chirality. Nat. Commun..

[41] Wu X., Teng F., Libera M. (2019). Functional Changes during Electron-Beam Lithography of Biotinylated Poly(Ethylene Glycol) Thin Films. ACS Macro Lett..

[42] Shaali M., Lara-Avila S., Dommersnes P., Ainla A., Kubatkin S., Jesorka A. (2015). Nanopatterning of Mobile Lipid Monolayers on Electron-Beam-Sculpted Teflon AF Surfaces. ACS Nano.

[43] Takei S., Oshima A., Oyama T.G., Ito K., Sugahara K., Kashiwakura M., Kozawa T., Tagawa S. (2014). Organic Solvent-Free Sugar-Based Transparency Nanopatterning Material Derived from Biomass for Eco-Friendly Optical Biochips Using Green Lithography. Biophotonics: Photonic Solutions for Better Health Care IV.

[44] Darko C., Botiz I., Reiter G., Breiby D.W., Andreasen J.W., Roth S.V., Smilgies D.-M., Metwalli E., Papadakis C.M. (2009). Crystallization in Diblock Copolymer Thin Films at Different Degrees of Supercooling. Phys. Rev. E.

[45] Botiz I., Freyberg P., Leordean C., Gabudean A.-M., Astilean S., Yang A.C.-M., Stingelin N. (2015). Emission Properties of MEH-PPV in Thin Films Simultaneously Illuminated and Annealed at Different Temperatures. Synth. Met..

[46] Botiz I., Grozev N., Schlaad H., Reiter G. (2008). The Influence of Protic Non-Solvents Present in the Environment on Structure Formation of Poly(γ-Benzyl-l-Glutamate) in Organic Solvents. Soft Matter.

[47] Botiz I., Astilean S., Stingelin N. (2016). Altering the Emission Properties of Conjugated Polymers: Emission Properties of Conjugated Polymers. Polym. Int..

[48] Leordean C., Marta B., Gabudean A.-M., Focsan M., Botiz I., Astilean S. (2015). Fabrication of Highly Active and Cost Effective SERS Plasmonic Substrates by Electrophoretic Deposition of Gold Nanoparticles on a DVD Template. Appl. Surf. Sci..

[49] Hawkes W., Huang D., Reynolds P., Hammond L., Ward M., Gadegaard N., Marshall J.F., Iskratsch T., Palma M. (2019). Probing the Nanoscale Organisation and Multivalency of Cell Surface Receptors: DNA Origami Nanoarrays for Cellular Studies with Single-Molecule Control. Faraday Discuss..

[50] Dague E., Jauvert E., Laplatine L., Viallet B., Thibault C., Ressier L. (2011). Assembly of Live Micro-Organisms on Microstructured PDMS Stamps by Convective/Capillary Deposition for AFM Bio-Experiments. Nanotechnology.

[51] Zhu S., Tang Y., Lin C., Liu X.Y., Lin Y. (2021). Recent Advances in Patterning Natural Polymers: From Nanofabrication Techniques to Applications. Small Methods.

[52] Humenik M., Winkler A., Scheibel T. (2021). Patterning of Protein-based Materials. Biopolymers.

[53] Liu W., Zhou Z., Zhang S., Shi Z., Tabarini J., Lee W., Zhang Y., Gilbert Corder S.N., Li X., Dong F. (2017). Precise Protein Photolithography (P3): High Performance Biopatterning Using Silk Fibroin Light Chain as the Resist. Adv. Sci..

[54] Kurland N.E., Dey T., Wang C., Kundu S.C., Yadavalli V.K. (2014). Silk Protein Lithography as a Route to Fabricate Sericin Microarchitectures. Adv. Mater..

[55] Pfirrmann S., Kirchner R., Lohse O., Guzenko V.A., Voigt A., Harder I., Kolander A., Schift H., Grützner G. (2016). mr-PosEBR: A Novel Positive Tone Resist for High Resolution Electron Beam Lithography and 3D Surface Patterning. Advances in Patterning Materials and Processes XXXIII.

[56] Wieberger F., Kolb T., Neuber C., Ober C.K., Schmidt H.-W. (2014). Nanopatterning with Tailored Molecules. Advances in Patterning Materials and Processes XXXI.

[57] Jiang J., Li X., Mak W.C., Trau D. (2008). Integrated Direct DNA/Protein Patterning and Microfabrication by Focused Ion Beam Milling. Adv. Mater..

[58] Sanchez-deAlcazar D., Romera D., Castro-Smirnov J., Sousaraei A., Casado S., Espasa A., Morant-Miñana M.C., Hernandez J.J., Rodríguez I., Costa R.D. (2019). Engineered Protein-Based Functional Nanopatterned Materials for Bio-Optical Devices. Nanoscale Adv..

[59] Chen S., Haehnle B., Van der Laan X., Kuehne A.J.C., Botiz I., Stavrinou P.N., Stingelin N. (2021). Understanding Hierarchical Spheres-in-Grating Assembly for Bio-Inspired Colouration. Mater. Horiz..

[60] Jeong H.-H., Lee J.-H., Lee C.-S., Jang H., Yang Y.-H., Kim Y.-H., Huh K.M. (2010). Fabrication of Selective Anti-Biofouling Surface for Micro/Nanopatterning of Proteins. Macromol. Res..

[61] Li G., Zhao X., Zhang L., Yang J., Cui W., Yang Y., Zhang H. (2020). Anisotropic Ridge/Groove Microstructure for Regulating Morphology and Biological Function of Schwann Cells. Appl. Mater. Today.

[62] MacNearney D., Mak B., Ongo G., Kennedy T.E., Juncker D. (2016). Nanocontact Printing of Proteins on Physiologically Soft Substrates to Study Cell Haptotaxis. Langmuir.

[63] Park S., Jackman J.A., Xu X., Weiss P.S., Cho N.-J. (2019). Micropatterned Viral Membrane Clusters for Antiviral Drug Evaluation. ACS Appl. Mater. Interfaces.

[64] Delamarche E., Pereiro I., Kashyap A., Kaigala G.V. (2021). Biopatterning: The Art of Patterning Biomolecules on Surfaces. Langmuir.

[65] Liu X., Kumar M., Calo’ A., Albisetti E., Zheng X., Manning K.B., Elacqua E., Weck M., Ulijn R.V., Riedo E. (2019). High-Throughput Protein Nanopatterning. Faraday Discuss..

[66] Lum W., Gautam D., Chen J., Sagle L.B. (2019). Single Molecule Protein Patterning Using Hole Mask Colloidal Lithography. Nanoscale.

[67] Du K., Park M., Ding J., Hu H., Zhang Z. (2017). Sub-10 Nm Patterning with DNA Nanostructures: A Short Perspective. Nanotechnology.

[68] Howorka S. (2013). DNA Nanoarchitectonics: Assembled DNA at Interfaces. Langmuir.

[69] Sun W., Shen J., Zhao Z., Arellano N., Rettner C., Tang J., Cao T., Zhou Z., Ta T., Streit J.K. (2020). Precise Pitch-Scaling of Carbon Nanotube Arrays within Three-Dimensional DNA Nanotrenches. Science.

[70] Kershner R.J., Bozano L.D., Micheel C.M., Hung A.M., Fornof A.R., Cha J.N., Rettner C.T., Bersani M., Frommer J., Rothemund P.W.K. (2009). Placement and Orientation of Individual DNA Shapes on Lithographically Patterned Surfaces. Nat. Nanotechnol..

[71] Weiger T.M., Hermann A. (2014). Cell Proliferation, Potassium Channels, Polyamines and Their Interactions: A Mini Review. Amino Acids.

[72] Blackiston D.J., McLaughlin K.A., Levin M. (2009). Bioelectric Controls of Cell Proliferation: Ion Channels, Membrane Voltage and the Cell Cycle. Cell Cycle.

[73] Hallstrom T.C., Nevins J.R. (2009). Balancing the Decision of Cell Proliferation and Cell Fate. Cell Cycle.

[74] Sato M., Kakui Y., Toya M. (2021). Tell the Difference Between Mitosis and Meiosis: Interplay Between Chromosomes, Cytoskeleton, and Cell Cycle Regulation. Front. Cell Dev. Biol..

[75] Fischer M., Dang C.V., DeCaprio J.A. (2018). Control of Cell Division. Hematology.

[76] Bagga S., Bouchard M.J., Noguchi E., Gadaleta M.C. (2014). Cell Cycle Regulation During Viral Infection. Cell Cycle Control.

[77] Coller H.A., Terjung R. (2019). Regulation of Cell Cycle Entry and Exit: A Single Cell Perspective. Comprehensive Physiology.

[78] Liu L., Michowski W., Kolodziejczyk A., Sicinski P. (2019). The Cell Cycle in Stem Cell Proliferation, Pluripotency and Differentiation. Nat. Cell Biol..

[79] John W., Baynes M., Dominiczak H. (2018). Medical Biochemistry.

[80] Lents N.H., Baldassare J.J. (2016). Cyclins and Cyclin-Dependent Kinases. Encyclopedia of Cell Biology.

[81] Yang H.W., Cappell S.D., Jaimovich A., Liu C., Chung M., Daigh L.H., Pack L.R., Fan Y., Regot S., Covert M. (2020). Stress-Mediated Exit to Quiescence Restricted by Increasing Persistence in CDK4/6 Activation. Elife.

[82] McIntosh J.R. (2016). Mitosis. Cold Spring Harb. Perspect. Biol..

[83] Ha G.-H., Breuer E.-K.Y. (2012). Mitotic Kinases and P53 Signaling. Biochem. Res. Int..

[84] Mitsushima M., Aoki K., Ebisuya M., Matsumura S., Yamamoto T., Matsuda M., Toyoshima F., Nishida E. (2010). Revolving Movement of a Dynamic Cluster of Actin Filaments during Mitosis. J. Cell Biol..

[85] Asbury C.L. (2017). Anaphase A: Disassembling Microtubules Move Chromosomes toward Spindle Poles. Biology.

[86] Weaver B.A.A., Cleveland D.W. (2005). Decoding the Links between Mitosis, Cancer, and Chemotherapy: The Mitotic Checkpoint, Adaptation, and Cell Death. Cancer Cell.

[87] Ebrahimi A., Ahmadi H., Ghasrodashti Z.P., Tanide N., Shahriarirad R., Erfani A., Ranjbar K., Ashkani-Esfahani S. (2021). Therapeutic Effects of Stem Cells in Different Body Systems, a Novel Method That Is yet to Gain Trust: A Comprehensive Review. Bosn. J. Basic Med. Sci..

[88] Yang B., Qiu Y., Zhou N., Ouyang H., Ding J., Cheng B., Sun J. (2017). Application of Stem Cells in Oral Disease Therapy: Progresses and Perspectives. Front. Physiol..

[89] Wei M., Li S., Le W. (2017). Nanomaterials Modulate Stem Cell Differentiation: Biological Interaction and Underlying Mechanisms. J. Nanobiotechnol..

[90] Mirzaei H., Sahebkar A., Sichani L.S., Moridikia A., Nazari S., Sadri Nahand J., Salehi H., Stenvang J., Masoudifar A., Mirzaei H.R. (2018). Therapeutic Application of Multipotent Stem Cells. J. Cell Physiol..

[91] Carpenedo R.L., McDevitt T.C. (2013). Stem Cells. Biomaterials Science.

[92] Romito A., Cobellis G. (2016). Pluripotent Stem Cells: Current Understanding and Future Directions. Stem Cells Int..

[93] Takahashi K., Yamanaka S. (2006). Induction of Pluripotent Stem Cells from Mouse Embryonic and Adult Fibroblast Cultures by Defined Factors. Cell.

[94] Zakrzewski W., Dobrzyński M., Szymonowicz M., Rybak Z. (2019). Stem Cells: Past, Present, and Future. Stem Cell Res. Ther..

[95] Raza Y., Salman H., Luberto C. (2021). Sphingolipids in Hematopoiesis: Exploring Their Role in Lineage Commitment. Cells.

[96] Hanna H., Mir L.M., Andre F.M. (2018). In Vitro Osteoblastic Differentiation of Mesenchymal Stem Cells Generates Cell Layers with Distinct Properties. Stem Cell Res. Ther..

[97] van der Sanden B., Dhobb M., Berger F., Wion D. (2010). Optimizing Stem Cell Culture. J. Cell. Biochem..

[98] Prokhorovich M.A., Lagar’kova M.A., Shilov A.G., Karamysheva T.V., Kiselyov S.L., Rubtsov N.B. (2007). Cultures of HESM Human Embryonic Stem Cells: Chromosomal Aberrations and Karyotype Stability. Bull. Exp. Biol. Med..

[99] Dakhore S., Nayer B., Hasegawa K. (2018). Human Pluripotent Stem Cell Culture: Current Status, Challenges, and Advancement. Stem Cells Int..

[100] Rao B., Zandstra P. (2005). Culture Development for Human Embryonic Stem Cell Propagation: Molecular Aspects and Challenges. Curr. Opin. Biotechnol..

[101] Sotiropoulou P.A., Perez S.A., Salagianni M., Baxevanis C.N., Papamichail M. (2006). Cell Culture Medium Composition and Translational Adult Bone Marrow-Derived Stem Cell Research. Stem Cells.

[102] Yao T., Asayama Y. (2017). Animal-Cell Culture Media: History, Characteristics, and Current Issues. Reprod. Med. Biol..

[103] Michaljaničová I., Slepička P., Heitz J., Barb R.A., Sajdl P., Švorčík V. (2015). Comparison of KrF and ArF Excimer Laser Treatment of Biopolymer Surface. Appl. Surf. Sci..

[104] Khare T., Oak U., Shriram V., Verma S.K., Kumar V. (2019). Biologically Synthesized Nanomaterials and Their Antimicrobial Potentials. Comprehensive Analytical Chemistry.

[105] Benninger R.K.P., Piston D.W. (2013). Two-Photon Excitation Microscopy for the Study of Living Cells and Tissues. Curr. Protoc. Cell Biol..

[106] Thorn K. (2016). A Quick Guide to Light Microscopy in Cell Biology. Mol. Biol. Cell..

[107] Daskalova A., Nathala C.S.R., Kavatzikidou P., Ranella A., Szoszkiewicz R., Husinsky W., Fotakis C. (2016). FS Laser Processing of Bio-Polymer Thin Films for Studying Cell-to-Substrate Specific Response. Appl. Surf. Sci..

[108] Inoué S., Pawley J.B. (2006). Foundations of Confocal Scanned Imaging in Light Microscopy. Handbook Of Biological Confocal Microscopy.

[109] Ragazzi M., Piana S., Longo C., Castagnetti F., Foroni M., Ferrari G., Gardini G., Pellacani G. (2014). Fluorescence Confocal Microscopy for Pathologists. Mod. Pathol..

[110] Slepička P., Michaljaničová I., Rimpelová S., Švorčík V. (2017). Surface Roughness in Action–Cells in Opposition. Mater. Sci. Eng. C.

[111] Ye C., Nikolov S.V., Calabrese R., Dindar A., Alexeev A., Kippelen B., Kaplan D.L., Tsukruk V.V. (2015). Self-(Un)Rolling Biopolymer Microstructures: Rings, Tubules, and Helical Tubules from the Same Material. Angew. Chem. Int. Ed..

[112] Dhand A.P., Galarraga J.H., Burdick J.A. (2021). Enhancing Biopolymer Hydrogel Functionality through Interpenetrating Networks. Trends Biotechnol..

[113] Augustine R., Hasan A., Dalvi Y.B., Rehman S.R.U., Varghese R., Unni R.N., Yalcin H.C., Alfkey R., Thomas S., Al Moustafa A.-E. (2021). Growth Factor Loaded in Situ Photocrosslinkable Poly(3-Hydroxybutyrate-Co-3-Hydroxyvalerate)/Gelatin Methacryloyl Hybrid Patch for Diabetic Wound Healing. Mater. Sci. Eng. C.

[114] Pradhan S., Moore K.M., Ainslie K.M., Yadavalli V.K. (2019). Flexible, Microstructured Surfaces Using Chitin-Derived Biopolymers. J. Mater. Chem. B.

[115] Estevam-Alves R., Ferreira P.H.D., Coatrini A.C., Oliveira O.N., Fontana C.R., Mendonca C.R. (2016). Femtosecond Laser Patterning of the Biopolymer Chitosan for Biofilm Formation. Int. J. Mol. Sci..

[116] Neto A.I., Vasconcelos N.L., Oliveira S.M., Ruiz-Molina D., Mano J.F. (2016). High-Throughput Topographic, Mechanical, and Biological Screening of Multilayer Films Containing Mussel-Inspired Biopolymers. Adv. Funct. Mater..

[117] Sarker M.D., Naghieh S., Sharma N.K., Ning L., Chen X. (2019). Bioprinting of Vascularized Tissue Scaffolds: Influence of Biopolymer, Cells, Growth Factors, and Gene Delivery. J. Healthc Eng..

[118] Sahu V., Marichi R.B., Singh G., Sharma R.K. (2017). Multifunctional, Self-Activating Oxygen-Rich Holey Carbon Monolith Derived from Agarose Biopolymer. ACS Sustain. Chem. Eng..

[119] Mao C., Xiang Y., Liu X., Cui Z., Yang X., Li Z., Zhu S., Zheng Y., Yeung K.W.K., Wu S. (2018). Repeatable Photodynamic Therapy with Triggered Signaling Pathways of Fibroblast Cell Proliferation and Differentiation To Promote Bacteria-Accompanied Wound Healing. ACS Nano.

[120] Cheng L., Yao B., Hu T., Cui X., Shu X., Tang S., Wang R., Wang Y., Liu Y., Song W. (2019). Properties of an Alginate-Gelatin-Based Bioink and Its Potential Impact on Cell Migration, Proliferation, and Differentiation. Int. J. Biol. Macromol..

[121] Neznalová K., Fajstavr D., Rimpelová S., Kasálková N.S., Kolská Z., Švorčík V., Slepička P. (2020). Honeycomb-Patterned Poly(L-Lactic) Acid on Plasma-Activated FEP as Cell Culture Scaffold. Polym. Degrad. Stab..

[122] Michaljaničová I., Slepička P., Rimpelová S., Slepičková Kasálková N., Švorčík V. (2016). Regular Pattern Formation on Surface of Aromatic Polymers and Its Cytocompatibility. Appl. Surf. Sci..

[123] Fajstavrová K., Rimpelová S., Fajstavr D., Švorčík V., Slepička P. (2020). PLLA Honeycomb-Like Pattern on Fluorinated Ethylene Propylene as a Substrate for Fibroblast Growth. Polymers.

[124] Hasturk O., Ermis M., Demirci U., Hasirci N., Hasirci V. (2018). Square Prism Micropillars Improve Osteogenicity of Poly(Methyl Methacrylate) Surfaces. J. Mater. Sci Mater. Med..

[125] Liu N., Zhou M., Zhang Q., Yong L., Zhang T., Tian T., Ma Q., Lin S., Zhu B., Cai X. (2018). Effect of Substrate Stiffness on Proliferation and Differentiation of Periodontal Ligament Stem Cells. Cell Prolif..

[126] Liu Q., Cen L., Yin S., Chen L., Liu G., Chang J., Cui L. (2008). A Comparative Study of Proliferation and Osteogenic Differentiation of Adipose-Derived Stem Cells on Akermanite and β-TCP Ceramics. Biomaterials.

[127] Jin R., Song G., Chai J., Gou X., Yuan G., Chen Z. (2018). Effects of Concentrated Growth Factor on Proliferation, Migration, and Differentiation of Human Dental Pulp Stem Cells in Vitro. J. Tissue Eng..

[128] Re F., Sartore L., Moulisova V., Cantini M., Almici C., Bianchetti A., Chinello C., Dey K., Agnelli S., Manferdini C. (2019). 3D Gelatin-Chitosan Hybrid Hydrogels Combined with Human Platelet Lysate Highly Support Human Mesenchymal Stem Cell Proliferation and Osteogenic Differentiation. J. Tissue Eng..

[129] Yang C.-Y., Huang W.-Y., Chen L.-H., Liang N.-W., Wang H.-C., Lu J., Wang X., Wang T.-W. (2021). Neural Tissue Engineering: The Influence of Scaffold Surface Topography and Extracellular Matrix Microenvironment. J. Mater. Chem. B.

[130] Rangappa N., Romero A., Nelson K.D., Eberhart R.C., Smith G.M. (2000). Laminin-Coated Poly(L-Lactide) Filaments Induce Robust Neurite Growth While Providing Directional Orientation. J. Biomed. Mater. Res..

[131] Inzana J.A., Olvera D., Fuller S.M., Kelly J.P., Graeve O.A., Schwarz E.M., Kates S.L., Awad H.A. (2014). 3D Printing of Composite Calcium Phosphate and Collagen Scaffolds for Bone Regeneration. Biomaterials.

[132] Simitzi C., Efstathopoulos P., Kourgiantaki A., Ranella A., Charalampopoulos I., Fotakis C., Athanassakis I., Stratakis E., Gravanis A. (2015). Laser Fabricated Discontinuous Anisotropic Microconical Substrates as a New Model Scaffold to Control the Directionality of Neuronal Network Outgrowth. Biomaterials.

[133] Antmen E., Ermis M., Demirci U., Hasirci V. (2019). Engineered Natural and Synthetic Polymer Surfaces Induce Nuclear Deformation in Osteosarcoma Cells. J. Biomed. Mater. Res. Part. B Appl. Biomater..

[134] Vrana N.E., Elsheikh A., Builles N., Damour O., Hasirci V. (2007). Effect of Human Corneal Keratocytes and Retinal Pigment Epithelial Cells on the Mechanical Properties of Micropatterned Collagen Films. Biomaterials.

[135] Gil E.S., Park S.-H., Marchant J., Omenetto F., Kaplan D.L. (2010). Response of Human Corneal Fibroblasts on Silk Film Surface Patterns. Macromol. Biosci..

[136] Li N., Chen G., Liu J., Xia Y., Chen H., Tang H., Zhang F., Gu N. (2014). Effect of Surface Topography and Bioactive Properties on Early Adhesion and Growth Behavior of Mouse Preosteoblast MC3T3-E1 Cells. ACS Appl. Mater. Interfaces.

[137] Nagata I., Kawana A., Nakatsuji N. (1993). Perpendicular Contact Guidance of CNS Neuroblasts on Artificial Microstructures. Development.

[138] Simitzi C., Ranella A., Stratakis E. (2017). Controlling the Morphology and Outgrowth of Nerve and Neuroglial Cells: The Effect of Surface Topography. Acta. Biomater..

[139] Schmalenberg K.E., Uhrich K.E. (2005). Micropatterned Polymer Substrates Control Alignment of Proliferating Schwann Cells to Direct Neuronal Regeneration. Biomaterials.

[140] Miller C., Jeftinija S., Mallapragada S. (2001). Micropatterned Schwann Cell–Seeded Biodegradable Polymer Substrates Significantly Enhance Neurite Alignment and Outgrowth. Tissue Eng..

[141] Wen X., Tresco P.A. (2006). Effect of Filament Diameter and Extracellular Matrix Molecule Precoating on Neurite Outgrowth and Schwann Cell Behavior on Multifilament Entubulation Bridging Devicein Vitro. J. Biomed. Mater. Res. A.

[142] Hsu S., Chen C.-Y., Lu P.S., Lai C.-S., Chen C.-J. (2005). Oriented Schwann Cell Growth on Microgrooved Surfaces. Biotechnol. Bioeng..

[143] Vleggeert-Lankamp C.L.A.M., Pêgo A.P., Lakke E.A.J.F., Deenen M., Marani E., Thomeer R.T.W.M. (2004). Adhesion and Proliferation of Human Schwann Cells on Adhesive Coatings. Biomaterials.

[144] Li W., Tang Q.Y., Jadhav A.D., Narang A., Qian W.X., Shi P., Pang S.W. (2015). Large-Scale Topographical Screen for Investigation of Physical Neural-Guidance Cues. Sci. Rep..

[145] Zhong C. (2020). Industrial-Scale Production and Applications of Bacterial Cellulose. Front. Bioeng. Biotechnol..

[146] Bottan S., Robotti F., Jayathissa P., Hegglin A., Bahamonde N., Heredia-Guerrero J.A., Bayer I.S., Scarpellini A., Merker H., Lindenblatt N. (2015). Surface-Structured Bacterial Cellulose with Guided Assembly-Based Biolithography (GAB). ACS Nano.

[147] Anderson J.M., Rodriguez A., Chang D.T. (2008). Foreign Body Reaction to Biomaterials. Semin. Immunol..

[148] Robotti F., Bottan S., Fraschetti F., Mallone A., Pellegrini G., Lindenblatt N., Starck C., Falk V., Poulikakos D., Ferrari A. (2018). A Micron-Scale Surface Topography Design Reducing Cell Adhesion to Implanted Materials. Sci. Rep..

[149] Ber S., Torun Köse G., Hasırcı V. (2005). Bone Tissue Engineering on Patterned Collagen Films: An in Vitro Study. Biomaterials.

[150] Murphy C.M., Haugh M.G., O’Brien F.J. (2010). The Effect of Mean Pore Size on Cell Attachment, Proliferation and Migration in Collagen–Glycosaminoglycan Scaffolds for Bone Tissue Engineering. Biomaterials.

[151] Anselme K. (2000). Osteoblast Adhesion on Biomaterials. Biomaterials.

[152] Yuan Y., Zhang P., Yang Y., Wang X., Gu X. (2004). The Interaction of Schwann Cells with Chitosan Membranes and Fibers in Vitro. Biomaterials.

[153] Li G., Zhao X., Zhao W., Zhang L., Wang C., Jiang M., Gu X., Yang Y. (2014). Porous Chitosan Scaffolds with Surface Micropatterning and Inner Porosity and Their Effects on Schwann Cells. Biomaterials.

[154] Gnavi S., Fornasari B., Tonda-Turo C., Laurano R., Zanetti M., Ciardelli G., Geuna S. (2015). The Effect of Electrospun Gelatin Fibers Alignment on Schwann Cell and Axon Behavior and Organization in the Perspective of Artificial Nerve Design. Int. J. Mol. Sci..

[155] Kalinina S., Gliemann H., López-García M., Petershans A., Auernheimer J., Schimmel T., Bruns M., Schambony A., Kessler H., Wedlich D. (2008). Isothiocyanate-Functionalized RGD Peptides for Tailoring Cell-Adhesive Surface Patterns. Biomaterials.

[156] binte M., Yusoff N.Z., Riau A.K., Yam G.H.F., binte Halim N.S.H., Mehta J.S. (2022). Isolation and Propagation of Human Corneal Stromal Keratocytes for Tissue Engineering and Cell Therapy. Cells.

[157] Huang J., Chen Y., Tang C., Fei Y., Wu H., Ruan D., Paul M.E., Chen X., Yin Z., Heng B.C. (2019). The Relationship between Substrate Topography and Stem Cell Differentiation in the Musculoskeletal System. Cell. Mol. Life Sci..

[158] Lanfer B., Seib F.P., Freudenberg U., Stamov D., Bley T., Bornhäuser M., Werner C. (2009). The Growth and Differentiation of Mesenchymal Stem and Progenitor Cells Cultured on Aligned Collagen Matrices. Biomaterials.

[159] Tay C.Y., Yu H., Pal M., Leong W.S., Tan N.S., Ng K.W., Leong D.T., Tan L.P. (2010). Micropatterned Matrix Directs Differentiation of Human Mesenchymal Stem Cells towards Myocardial Lineage. Exp. Cell Res..

[160] Younesi M., Islam A., Kishore V., Anderson J.M., Akkus O. (2014). Tenogenic Induction of Human MSCs by Anisotropically Aligned Collagen Biotextiles. Adv. Funct. Mater..

[161] Seo C.H., Jeong H., Feng Y., Montagne K., Ushida T., Suzuki Y., Furukawa K.S. (2014). Micropit Surfaces Designed for Accelerating Osteogenic Differentiation of Murine Mesenchymal Stem Cells via Enhancing Focal Adhesion and Actin Polymerization. Biomaterials.

[162] Hashemzadeh H., Allahverdi A., Ghorbani M., Soleymani H., Kocsis Á., Fischer M.B., Ertl P., Naderi-Manesh H. (2019). Gold Nanowires/Fibrin Nanostructure as Microfluidics Platforms for Enhancing Stem Cell Differentiation: Bio-AFM Study. Micromachines.

[163] Zhang C., Yuan H., Liu H., Chen X., Lu P., Zhu T., Yang L., Yin Z., Heng B.C., Zhang Y. (2015). Well-Aligned Chitosan-Based Ultrafine Fibers Committed Teno-Lineage Differentiation of Human Induced Pluripotent Stem Cells for Achilles Tendon Regeneration. Biomaterials.

[164] Evans N.D., Minelli C., Gentleman E., LaPointe V., Patankar S.N., Kallivretaki M., Chen X., Roberts C.J., Stevens M.M. (2009). Substrate Stiffness Affects Early Differentiation Events in Embryonic Stem Cells. Eur. Cells Mater..

[165] Shukla A., Slater J.H., Culver J.C., Dickinson M.E., West J.L. (2016). Biomimetic Surface Patterning Promotes Mesenchymal Stem Cell Differentiation. ACS Appl. Mater. Interfaces.

[166] Metavarayuth K., Sitasuwan P., Zhao X., Lin Y., Wang Q. (2016). Influence of Surface Topographical Cues on the Differentiation of Mesenchymal Stem Cells in Vitro. ACS Biomater. Sci. Eng..

[167] Bhadriraju K., Yang M., Alom Ruiz S., Pirone D., Tan J., Chen C.S. (2007). Activation of ROCK by RhoA Is Regulated by Cell Adhesion, Shape, and Cytoskeletal Tension. Exp. Cell Res..

[168] Wang Y.-K., Yu X., Cohen D.M., Wozniak M.A., Yang M.T., Gao L., Eyckmans J., Chen C.S. (2012). Bone Morphogenetic Protein-2-Induced Signaling and Osteogenesis Is Regulated by Cell Shape, RhoA/ROCK, and Cytoskeletal Tension. Stem Cells Dev..

[169] Gu S.R., Kang Y.G., Shin J.W., Shin J.-W. (2017). Simultaneous Engagement of Mechanical Stretching and Surface Pattern Promotes Cardiomyogenic Differentiation of Human Mesenchymal Stem Cells. J. Biosci. Bioeng..

[170] Ding H., Zhong J., Xu F., Song F., Yin M., Wu Y., Hu Q., Wang J. (2017). Establishment of 3D Culture and Induction of Osteogenic Differentiation of Pre-Osteoblasts Using Wet-Collected Aligned Scaffolds. Mater. Sci. Eng. C.

[171] Tuleuova N., Lee J.Y., Lee J., Ramanculov E., Zern M.A., Revzin A. (2010). Using Growth Factor Arrays and Micropatterned Co-Cultures to Induce Hepatic Differentiation of Embryonic Stem Cells. Biomaterials.

[172] Williams D.F. (2019). Challenges With the Development of Biomaterials for Sustainable Tissue Engineering. Front. Bioeng. Biotechnol..

[173] Shahbazi M., Jäger H. (2021). Current Status in the Utilization of Biobased Polymers for 3D Printing Process: A Systematic Review of the Materials, Processes, and Challenges. ACS Appl. Bio. Mater..

[174] Fernandes C., Moura C., Ascenso R.M.T., Amado S., Alves N., Pascoal-Faria P., Yasa E., Mhadhbi M., Santecchia E. (2020). Comprehensive Review on Full Bone Regeneration through 3D Printing Approaches. Design and Manufacturing.

